# VCP modulation ameliorates pathological features in C9orf72 models

**DOI:** 10.1038/s41419-026-08856-1

**Published:** 2026-05-17

**Authors:** Veronica Ferrari, Barbara Tedesco, Marta Cozzi, Paola Pramaggiore, Maria Cristina Gagliani, Rocio Magdalena, Laura Cornaggia, Elena Casarotto, Marta Chierichetti, Ali Mohamed, Maria Brodnanovà, Carmelo Milioto, Margherita Piccolella, Mariarita Galbiati, Valeria Crippa, Alessandro Provenzani, Katia Cortese, Paola Rusmini, Riccardo Cristofani, Angelo Poletti

**Affiliations:** 1 Dipartimento di Scienze Farmacologiche e Biomolecolari “Rodolfo Paoletti” (DiSFeB), Università degli Studi di Milano, Milan, Italy; 2 Department of Experimental Medicine (DIMES), University of Genoa, Genova, Italy; 3 Department of Cellular, Computational and Integrative Biology (CIBIO) - University of Trento, Povo, Italy

**Keywords:** Amyotrophic lateral sclerosis, Cell death in the nervous system

## Abstract

Amyotrophic lateral sclerosis (ALS) and frontotemporal dementia (FTD) are devastating neurodegenerative diseases linked by similar pathological mechanisms, which, in some familial forms, may be associated with the same genetic alterations. Among them, the most common is the *C9ORF72* (C9) mutation. The *C9* mutation consists in an aberrant expansion of the hexanucleotide repeat (G_4_C_2_)_n_ that leads to the production and accumulation of toxic dipeptide repeat proteins (DPRs). Some of these C9-DPRs contribute to neuronal dysfunction and degeneration through different mechanisms. One of these involves alterations in the protein quality control (PQC) system, specifically in the autophagy-lysosomal pathway. Valosin-containing protein (VCP) is a critical component of the PQC system, assisting the degradation of misfolded proteins and damaged organelles and the maintenance of cellular homeostasis. In this study, we investigated the role of VCP in modulating pathological features associated with C9 mutation. Using neuronal cell models, we demonstrated that VCP overexpression significantly reduced C9-DPRs levels. This reduction is mediated by mechanisms involving both the ubiquitin-proteasome system (UPS) and autophagy. Additionally, we also observed that C9-DPRs induce lysosomal damage, which is counteracted by VCP overexpression, as indicated by decreased galectin-3 puncta and restored lysosomal pH. We then pharmacologically activated VCP-mediated clearance through SMER28, increasing the clearance of the most toxic DPR, the polyPR. We also determined that in this model, SMER28 activity is mediated by the UPS and is associated with the mitigation of DPR-induced lysosome damage. Additionally, using motor neurons derived from induced pluripotent stem cells (iPSC-MNs) from *C9-ALS* mutation carriers, we demonstrated that SMER28 treatment significantly decreased polyGA levels, a marker for C9-DPR accumulation. Moreover, SMER28 rescued C9-MNs commitment to differentiation and the alteration in the expression of autophagy-related genes. Taken together, our findings strongly support VCP as a modulator of C9 pathology and highlight its potential as a therapeutic target.

## Introduction

Amyotrophic lateral sclerosis (ALS) and frontotemporal dementia (FTD) are two fatal neurodegenerative diseases (NDs) characterized by the loss of selected neuronal populations. In ALS, mainly upper cortical and lower bulbar and spinal cord motor neurons (MNs) are primarily affected, while in FTD, progressive neuronal loss occurs in the frontal and temporal lobes. Different pathological mechanisms concur to neuronal death, such as oxidative stress, organelle dysfunction, impairment of axonal transport, excitotoxicity, protein aggregation, alterations in protein quality control (PQC) system, endoplasmic reticulum stress, alterations in RNA processing, and neuroinflammation [1, 2]. Notably, ALS and FTD share several pathophysiological mechanisms. Indeed, some ALS cases present typical characteristics of FTD and vice versa*,* highlighting the close connection between these two NDs [3]. Both diseases are characterized by TAR DNA-binding protein 43 (TDP-43) mislocalization, phosphorylation, cleavage, and aggregation, known as TDP-43 pathology. Moreover, ALS and FTD share the presence of protein inclusions related to a high propensity of disease-related proteins to aggregate and/or to failure of the PQC system.

A high percentage of ALS and FTD cases has been associated with a mutation in the *C9ORF72 (C9)* gene (C9-ALS or C9-FTD) [4, 5]. *C9* expression is implicated in different pathways, including nucleocytoplasmic import, stress granule formation and degradation, endosomal trafficking, axon growth, and autophagy regulation [6–11], reviewed in [12]. *C9* mutation consists in the abnormal expansion of the hexanucleotide sequence (G_4_C_2_ or the antisense C_4_G_2_) present in the first intron of the *C9* gene. Patients present up to thousands of these repeats, while physiologically the repeat can be found no more than 2 to 23 times [4, 5]. The (G_4_C_2_)_n_ expansion is associated with loss of function causing haploinsufficiency on one side, and gain of function on the other [13–16]. Gain of function relates either to the formation of RNA foci in the nucleus triggered by the abnormal RNA structures, or by an unconventional “repeat-associated non-ATG” translation (RAN-translation) mechanism that, on the RNAs encoded by both DNA strands in the *C9 locus*, produces 5 dipeptide repeat proteins (DPRs): polyGA, polyGP, polyGR, polyPR, and polyPA [17]. DPRs accumulate in the cytoplasm and/or nucleus of neurons and glial cells and are toxic since they sequester fundamental proteins and alter organelle structures (e.g., lysosomes) [18]. Of note, the *C9* mutation was associated with a decrease in lysosomal trafficking and activity in MNs differentiated from induced pluripotent stem cells (iPSCs) [18]. Moreover, the mutation alters the nuclear import of transcription factors that regulate lysosome biogenesis and clearance [19]. Lysosomal alterations result in detrimental variations of cell homeostasis (reviewed in [20]).

Thus, the removal or the prevention of C9-DPRs formation by promoting the modulation of the PQC system could ameliorate the pathological features associated with the *C9* mutation. The PQC system comprises several chaperone proteins and two major degradation systems, the ubiquitin-proteasome system (UPS) and autophagy. Valosin-containing protein (VCP) is an AAA^+^ protein involved in many pathways of the PQC system [21]. It segregates proteins from protein aggregates, complexes, or membranes and induces their clearance through the UPS. Upon binding to co-factors and adaptors, VCP regulates the degradation of proteins cooperating in different mechanisms, including the clearance of altered proteins or damaged organelles [22, 23]. VCP has also been found strictly implicated in autophagy regulation [24]. Mutations in *VCP* trigger toxic cellular alterations typical of a dysfunctional PQC system, underlining its essential role in proteostasis [25, 26]. Some *VCP* mutations have been shown to have increased activity; thus, different studies showed the rescue of PQC alteration or the increase of protein aggregation in VCP models by using VCP inhibitors or recently also antisense oligonucleotides (ASOs) [27, 28]. Differently, several studies on different NDs unrelated to *VCP* mutations have demonstrated that exogenous expression of VCP or modulation of its activity can reduce the accumulation of aggregation-prone proteins associated with these NDs. Indeed, VCP expression promotes autophagy-mediated clearance of tau aggregates, associated with FTD or Alzheimer’s disease, or the degradation of mutant SOD1 aggregates present in one of the various familial forms of ALS (fALS) [29, 30]. Also, different studies showed that the pharmacological activation of VCP enhances the clearance of mutated proteins such as ataxin 3 and huntingtin in NDs [31] and the rescue of TDP-43 aggregation in models of multisystem proteinopathies.

Interestingly, VCP was found to be involved in *C9* mutation models. VCP was shown to be sequestered by polyGA [32], modifying its localization and impairing its correct functioning. This suggestes VCP involvement in DPR clearance.

Thus, here we investigated whether VCP modulation could be beneficial by rescuing C9 pathological features and by promoting the decrease of DPR accumulation and the clearance of altered organelles, in particular lysosomes.

Starting from these findings, we genetically and pharmacologically modulated VCP in C9 cellular models to determine its protective activity against the pathogenic mechanism involved in C9 pathology. We first showed that VCP overexpression results in a significant decrease in the accumulation of C9-DPRs. We then modulated VCP-mediated aggregate clearance through UPS and autophagy using SMER28, a VCP activator in these specific pathways. Interestingly, SMER28 treatment reduced polyPR clearance, confirming VCP involvement and highlighting a specific VCP-mediated pathway.

We then characterized C9-induced lysosomal damage in our in vitro model, showing that VCP upregulation prevents this damage. Finally, we studied VCP modulation in MNs differentiated from iPSCs derived from an ALS-*C9* patient, a more relevant model to study the disease. We pharmacologically induced VCP activation using SMER28, which led to a decrease in polyGA protein levels and the rescue of C9-MNs commitment to differentiation. Altogether, these findings reinforced the role of VCP in the pathology and its potential target for a therapeutic approach in C9 related pathology.

## Results

### Characterization of lysosomal damage induced by the *C9orf72* mutation in a neuronal model

**Fig. 1:**
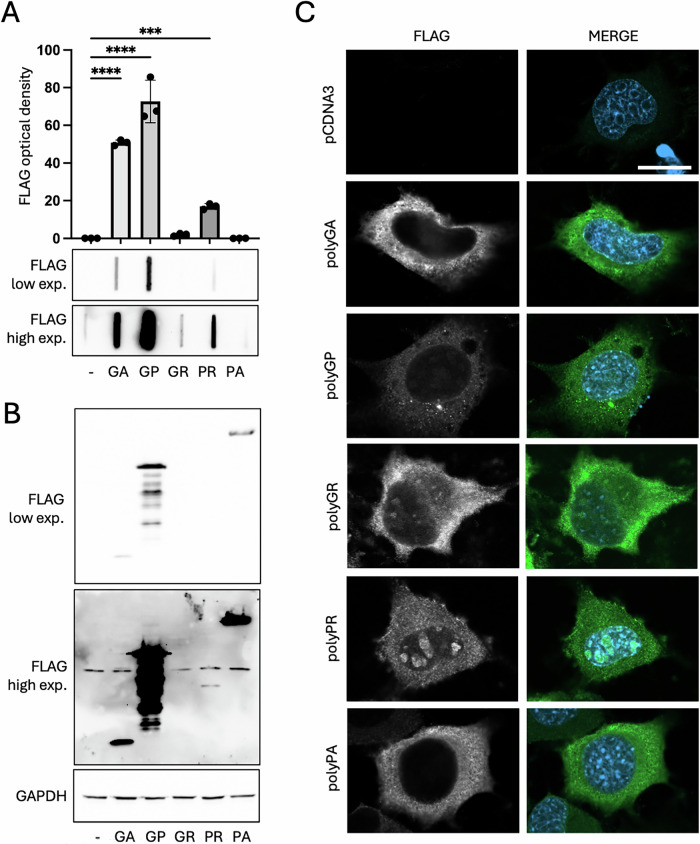
Characterization of DPR aggregates in NSC34 cells. **A** Graph (upper inset) of the filter retardation assay (FRA) of PBSextracts from NSC34 cells overexpressing each single DPR tagged with FLAG (lower inset), (One-way ANOVA followed by Fisher’s LSD test; ***p < 0.001; ****p < 0.0001). **B** Representative western blot (WB) of SDS-extracts from NSC34 overexpressing each single DPR marked with FLAG-antibody. GAPDH is used as a loading control. **C** Immunofluorescence (IF) analysis (63x magnification) on NSC34 overexpressing all the single FLAG-DPRs. Scale bar = 10 µm.

*C9* mutation has been demonstrated to impact the autophagy-lysosomal pathway (ALP) in human cells and *Drosophila melanogaster* models, resulting in a decrease of lysosomal enzymatic activity, an enlargement of lysosomes, and an increase of multilamellar bodies (MLBs) [18, 19]. To determine how the different DPRs are responsible for these alterations in our model, we studied the impact of C9-DPR expression on lysosomes in immortalized motoneuronal NSC34 cells expressing each single C9-DPR. We evaluated the presence of C9-DPR aggregates by filter retardation assay (FRA) and immunofluorescence (IF) analyses (Fig. 1A, C). Specifically, polyGA, polyGP, polyGR, and polyPR presented different levels of high molecular weight (HMW) insoluble species visible through FRA analysis. PolyGP insoluble species detected by FRA showed higher levels than the other C9-DPRs, apparently in contrast with our previous report [33]. A possible explanation is related to the use of different reagents with an improvement of C9-DPRs expression and detection in NSC34 cells (Supplementary Fig. 1A). Therefore, we compared the effect of the two different transfectant reagents (lipofectamine (LIPO) and LIPO3000) on polyGA, polyGP, and polyPR accumulation. Indeed, transfection of plasmids encoding polyGP and polyPR with LIPO3000 resulted in an increased accumulation of these DPRs in FRA compared to LIPO, probably due to a more efficient transfection. On the contrary, polyGA levels remained unchanged, which is in line with its low tendency to aggregate. The presence of polyGA, polyGP, and polyPR was confirmed by western blot (WB), while polyGR was present in lower protein levels due to its unstable nature. Conversely, polyPA, which is characterized by undetectable HMW insoluble species, displayed high protein levels in WB, confirming its highly soluble nature (Fig. 1A, B). C9-DPRs localization was detected by IF: polyGA, polyGP, and polyPA presented a cytoplasmatic localization with few visible aggregates; the two most toxic DPRs containing the arginine residue, polyGR and polyPR, were characterized by mixed nucleus/cytoplasm localization with large nuclear aggregates (Fig. 1C).

**Fig. 2:**
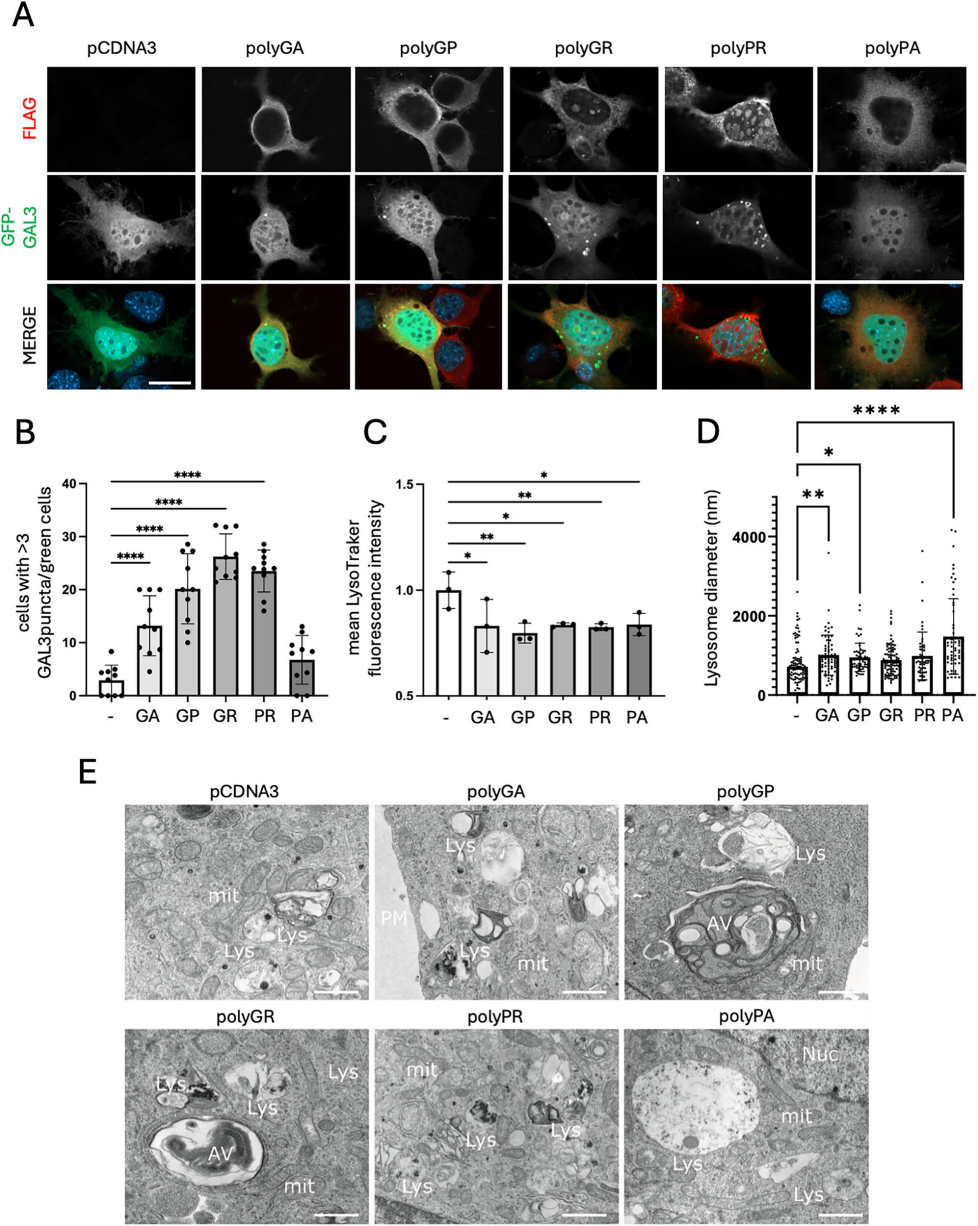
DPRs induce lysosomal alterations in NSC34 cells. **A** IF analysis (63x magnification) on NSC34 cells overexpressing FLAG-DPRs and EGFP-LGALS3 (GFP-GAL3). FLAG-DPRs were stained with an anti-FLAG antibody (red), and nuclei were stained with DAPI (blue). Scale bar = 10 µm. **B** The bar graph represents the quantification of percentage of cells with >3 EGFP-LGALS3 puncta after transfection with each single DPR, over 9 independent biological samples for each condition (*n* = 9) (One-way ANOVA followed by Fisher’s LSD test; ****p < 0.0001). **C** The bar graph represents the quantification of the mean LysoTracker fluorescence intensity measured by cytofluorimetric analysis performed on NSC34 cells overexpressing FLAG-DPRs and labeled with LysoTracker Green. Mean fluorescence intensity was measured (*n* = 4) (One-way ANOVA followed by Fisher’s LSD test; *p < 0.05, **p < 0.01). **D** The bar graph represents the quantification of lysosome diameter (nm) performed on NSC34 cells overexpressing FLAG-DPRs (*n* = 10) (One-way ANOVA followed by Kruskal-Wallis test; *p < 0.05, **p < 0.01, ****p < 0.001). **E** Representative TEM micrographs showing lysosomal ultrastructure (Lys) in NSC34 cells overexpressing FLAG-DPRs. Autophagic vacuoles (AV), nucleus (nuc), plasma membrane (PM), and mitochondria (mit) are also indicated. Scale bar = 1 µm.

We next assessed the impact of each C9-DPR expressed in NSC34 cells on lysosomes. Lysosomal damage was studied by analyzing galectin-3 (LGALS3 or GAL3) puncta, a marker of lysosomal membrane permeabilization. GAL3 is normally diffused in the cytoplasm, but when lysosome membrane permeabilization occurs, it binds to lysosome membranes, and this results in its punctate distribution. Results showed an increase in GAL3 puncta in the presence of all DPRs except for polyPA (Fig. 2A, B). Lysosomal impairment was then confirmed by LysoTracker analysis, which revealed an alteration of lysosomal pH induced by all five C9-DPRs expression compared with the control (Fig. 2C). Moreover, NSC34 expressing C9-DPRs were analyzed through transmission electron microscopy (TEM). By measuring lysosomal diameter in Fig. 2D, we found an increase in lysosomal size in cells expressing the C9-DPRs, which was highly significant for polyGA, polyGP, and polyPA (Fig. 2D). Of note, TEM analysis revealed a mixed population of altered lysosomes associated with C9-DPR expression (Fig. 2E). Indeed, cells expressing polyGP and polyGR were characterized by the accumulation of autophagic vacuoles, while cells expressing polyPR presented lysosomes containing electron-dense material, which can be hypothesized to be protein aggregates.

Altogether, these data indicate a critical and complex lysosomal impairment induced by the expression of each C9-DPR.

### C9-DPRs alter TFEB and TFE3 localization

**Fig. 3:**
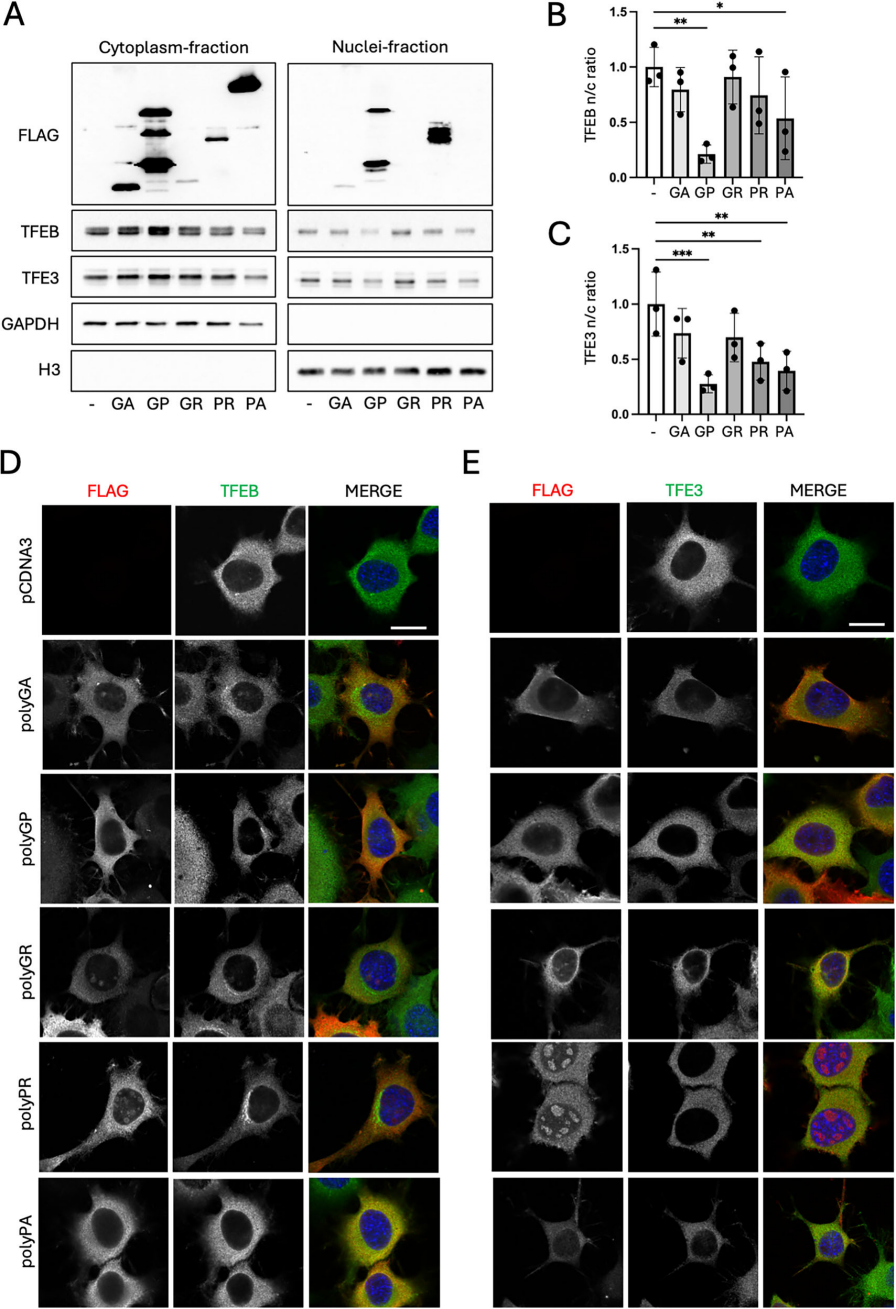
DPRs alter TFEB and TFE3 nuclear/cytoplasmatic ratio. **A** Representative WB of cytosol and nuclei fractions of NSC34 cells expressing each single DPR. To visualize FLAG-DPRs, an anti-FLAG antibody was used. To visualize TFEB or TFE3, anti-TFEB or TFE3 antibodies were used. GAPDH and histone H3 were used as loading controls for the cytoplasmic and nuclear fractions, respectively. **B** Quantification of TFEB nuclear/cytoplasmic fraction is represented in Fig. 3A. The bar graph represents the ratio of the mean relative optical density quantification of nuclear to cytoplasmic TFEB detected by WB using an anti-TFEB antibody (One-way ANOVA followed by Fisher’s LSD test; *p < 0.05, **p < 0.01). **C** Quantification of TFE3 nuclear/cytoplasmic fraction is represented in Fig. 3A. The bar graph represents the ratio of the mean relative optical density quantification of nuclear to cytoplasmic TFE3 detected by WB using an anti-TFE3 antibody. (One-way ANOVA followed Fisher’s LSD test; **p < 0.01, ***p < 0.001). **D** Fluorescence microscopy analysis (63x magnification) of NSC34 cells expressing each single DPR. TFEB was visualized using an anti-TFEB antibody (green), DPRs were visualized using an anti-FLAG antibody (red), and nuclei were stained with DAPI (blue). Scale bar = 10 μm. **E** Fluorescence microscopy analysis (63x magnification) of NSC34 cells expressing each single DPR. TFE3 was visualized using an anti-TFE3 antibody (green), DPRs were visualized using an anti-FLAG antibody (red), and nuclei were stained with DAPI (blue). Scale bar = 10 μm.

We and others have previously described that the persistence of damaged lysosomes activates their clearance through lysophagy and enhances lysosomal biogenesis at the transcriptional level [25, 34]. The key regulators of these pathways are the transcription factor EB (TFEB) and Transcription Factor Binding To IGHM Enhancer 3 (TFE3), generally believed to exert identical functions in this context [35]. Hence, we studied the nuclear translocation of the activated forms of TFEB and TFE3 in NSC34 cells expressing each single C9-DPR (Fig. 3).

We observed that all C9-DPRs impact the distribution of the two transcription factors. Particularly, polyGP and polyPA induced a significant decrease in the nuclear/cytoplasmatic ratio of TFEB and TFE3, while polyGR significantly affected only the TFE3 nuclear/cytoplasmatic ratio (Fig. 3B, C). In contrast, polyGA and polyPR did not seem to alter TFEB and TFE3 levels and partitioning (Fig. 3A, C). Interestingly, IF analysis showed an accumulation of TFEB in the perinuclear area in the presence of polyGA, polyPR, and, even if to a lower extent, polyGR (Fig. 3D). As for TFE3 localization, no alterations were detected by IF in NSC34 cells expressing the various C9-DPRs (Fig. 3E).

Alteration of TFEB/TFE3 nuclear levels suggested a dysregulation of Coordinated Lysosomal Expression and Regulation (CLEAR) gene expression. Thus, we performed a functional study of TFEB/TFE3 alterations by investigating the expression of autophagy-related genes (Supplementary Fig. 1B). In contrast with our hypothesis, we did not note any differences in the genes considered. However, we cannot exclude that more experiments at different time points or by stressing the mechanisms could show an outcome of TFEB/TFE3 dysregulation.

### VCP overexpression decreases the HMW insoluble species formed by the C9-DPRs

**Fig. 4:**
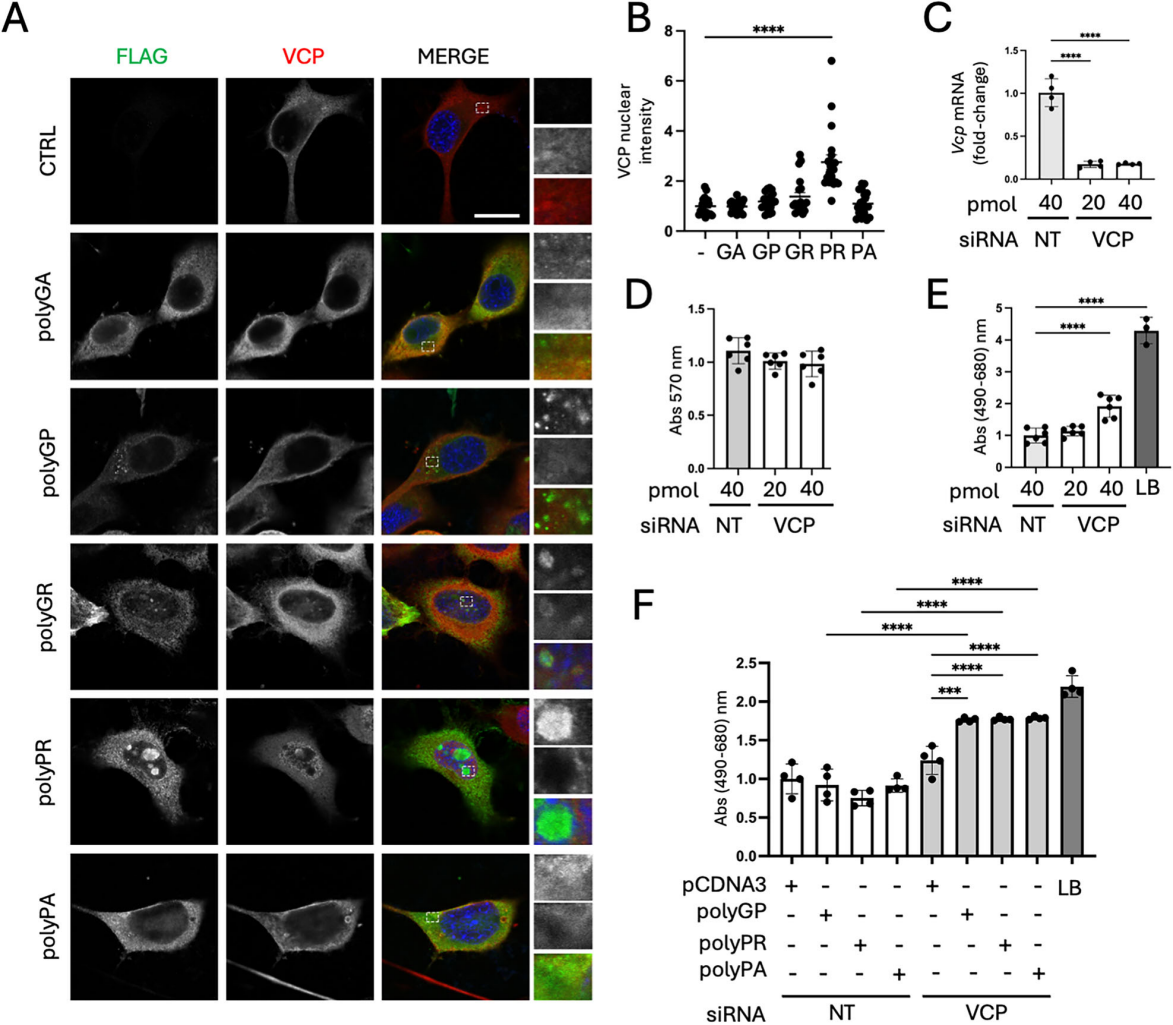
VCP and C9-DPRs interaction. **A** IF analysis (63x magnification) on NSC34 cells overexpressing FLAG-DPRs. FLAG-DPRs were stained with an anti-FLAG antibody (green), endogenous VCP was stained with an anti-VCP antibody (red), and nuclei were stained with DAPI (blue). Scale bar = 10 µm. **B** Quantification of VCP nuclear intensity; the fields were randomly selected, and at least 25 cells were analyzed for each condition (one-way ANOVA with Fisher’s LSD test; ****p < 0.0001) (**B**) NSC34 cells transfected with siRNA against VCP or non-targeting (NT). RT-qPCR for *Vcp* mRNA normalized with *Rplp0* mRNA levels. Data are means ∓SD of 4 independent samples. NSC34 transfected with siRNA against VCP (One-way ANOVA followed by Fisher’s LSD test; ****p < 0.0001). **C** Cell viability MTT assay on NSC34 cells treated with siRNA-VCP at 20 or 40 pmol/ml. **D** LDH cytotoxicity assay on NSC34 cells treated with siRNA-VCP at 20 or 40 pmol/ml. Lysis buffer (LB) was used to evaluate the maximum LDH release (One-way ANOVA followed by Fisher’s LSD test; ****p < 0.0001). **E** LDH assay on NSC34 cells transfected with siRNA-VCP/NT and plasmids expressing polyGP, polyPR, polyPA (One-way ANOVA followed by Fisher’s LSD test; ***p < 0.001, ****p < 0.0001).

As mentioned above, VCP is an important regulator of proteostasis, since it concurs in the degradation of nonfunctional, altered, and aberrant proteins through UPS or autophagy. Moreover, VCP is essential for the degradation of a subset of damaged lysosomes and other organelles through autophagy [24]. VCP is known to interact with polyGA, and it was suggested as an effector of its clearance. We analyzed VCP behavior in NSC34 cells expressing C9-DPRs and observed that, in basal conditions, VCP is mainly diffusely distributed in the cytoplasm. Similarly, VCP remained diffusely localized in the presence of polyGA, polyGP, and polyPA aggregates, whereas both polyGR and polyPR nuclear aggregates altered VCP localization, causing its relocalization to the nucleus (Fig. 4A). By quantifying VCP nuclear signal levels in comparison to the control condition, we noted a significant increase in VCP nuclear accumulation associated with polyPR expression, while for polyGR, VCP nuclear accumulation tended to increase, but did not reach a significant value (Fig. 4B). On the contrary, there seem to be no alterations in the total protein amount of VCP evaluated by WB (Supplementary Fig. 2A).

**Fig. 5:**
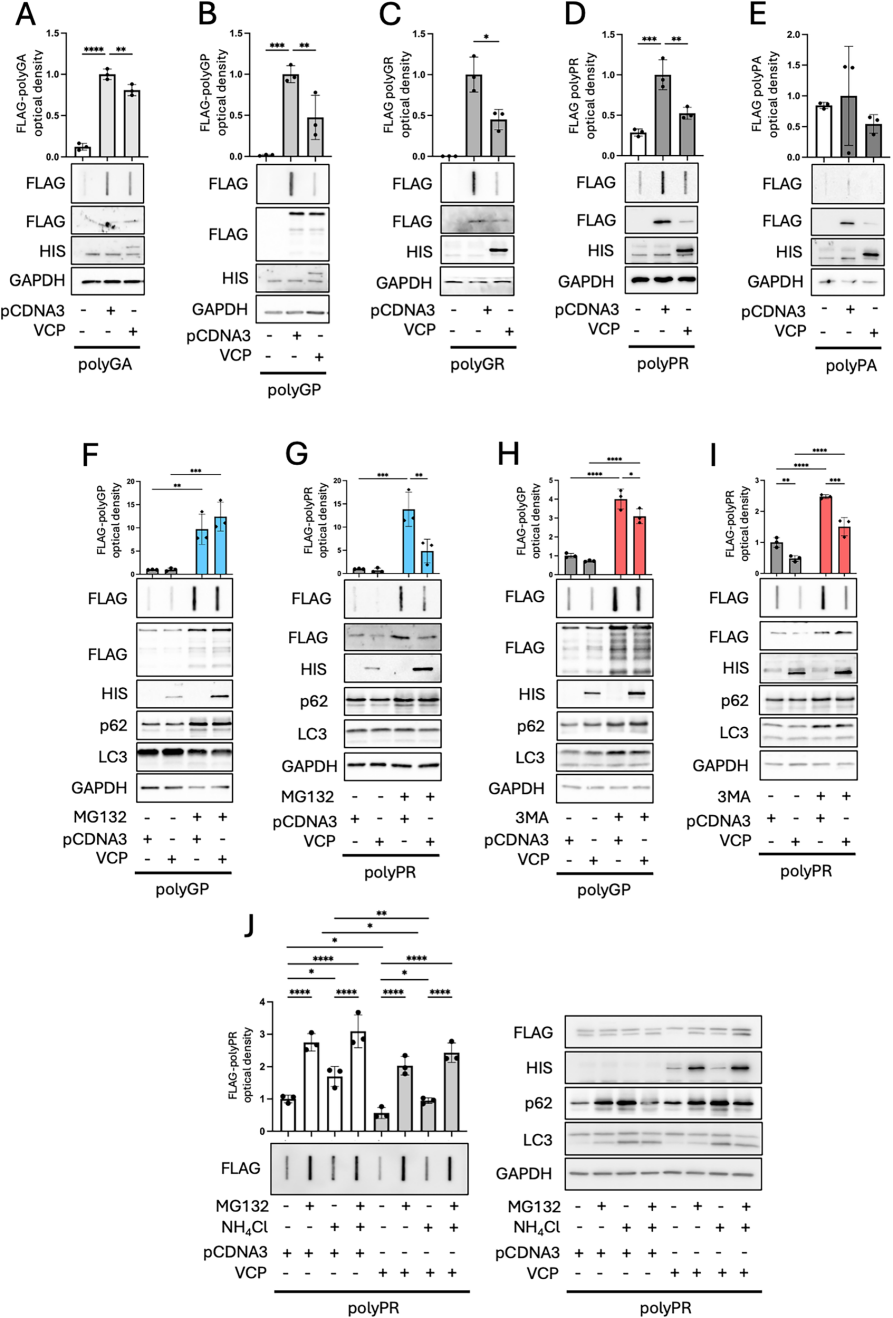
VCP overexpression in NSC34 expressing C9-DPRs. **A**–**E** NSC34 overexpressing FLAG-DPRs and pCDNA3/HIS-VCP. Graph (upper inset) of FRA of each DPR visualized with anti-FLAG antibody (middle inset). A representative WB (lower inset) of DRPs, marked with anti-FLAG antibody, and VCP marked with anti-HIS. GAPDH was used as a loading control. **F**, **G** NSC34 overexpressing FLAG-DPRs and pCDNA3/HIS-VCP and treated with proteasome inhibitor MG132. GAPDH was used as a loading control. **H**, **I** NSC34 overexpressing FLAG-DPRs and pCDNA3/HIS-VCP and treated with autophagy inhibitor 3-MA. **J** NSC34 overexpressing FLAG-polyPR and pCDNA3/HIS-VCP and treated with MG132 and/or NH_4_Cl. Graph (upper inset) of the FRA of each DPR visualized with anti-FLAG antibody (middle inset). A representative WB (lower inset) of DRPs, marked with anti-FLAG antibody, and VCP marked with anti-HIS. To visualize degradation system markers, p62 and LC3, anti-p62/LC3 antibodies were used. GAPDH was used as a loading control. For all the statistical analysis, one-way ANOVA followed Fisher’s LSD test was used; *p < 0.05, **p < 0.01, ***p < 0.001, ****p < 0.0001).

To evaluate the importance of VCP in cell response to C9-DPR aggregates, we silenced VCP in NSC34 cells. We firstly identified the condition of use of the siRNA against VCP by testing the efficiency and the impact on viability of different concentrations (Fig. 4C-E). We then overexpressed polyGP, polyPR, and polyPA, hypothesizing that VCP silencing would increase their aggregate levels. However, we failed to evaluate this parameter due to very high toxicity of C9-DPRs in the absence of VCP. We measured toxicity with the LDH assay (Fig. 4F). These data strongly support the relevance of VCP in protecting against C9-DPRs toxicity. Thus, we hypothesize that a positive modulation of VCP could decrease DPRs levels. We overexpressed VCP in NSC34 cells expressing each DPR. Indeed, we observed a decrease in polyGA, polyGP, polyPR, and polyGR HMW insoluble species upon VCP overexpression, as shown in WB and FRA results (Fig. 5A–D). A decrease in polyPA levels was not detectable, as it does not form HMW insoluble species by FRA analysis (Fig. 5E).

Knowing the multifaceted role of VCP in the PQC, we characterized how VCP mediates the degradation of C9-DPRs. We focused on polyGP and polyPR as they were the most affected by VCP modulation. We firstly performed an immunoprecipitation study using an anti-FLAG antibody on NSC34 cells expressing the FLAG-tagged polyGP and polyPR (Supplementary Fig. 2B). We observed the presence of VCP on the immunoprecipitated polyGP, demonstrating a direct interaction between them. We failed to observe a direct interaction between VCP and polyPR in the condition tested. Then, we pharmacologically inhibited the UPS using MG132 (Fig. 5F, G) or autophagy using 3-MA (Fig. 5H, I). The efficacy of these treatments was confirmed by the accumulation of Sequestosome-1 (SQSTM1 or p62) and the activation of Microtubule-associated protein 1 A/1B-light chain 3 (MAP1LC3 or LC3), autophagy markers (Fig. 5F–I). Notably, we observed a significant increase in polyGP and polyPR levels upon proteasome and autophagy inhibition. We had previously demonstrated the impact of inhibiting these degradation systems on polyGP; however, an increase in polyPR levels was not detected in that case [33]. Thus, we assumed that the change in transfection reagent further influenced the system, amplifying the differences between the tested conditions. As shown in Supplementary Fig. 3A, B, transfection with LIPO3000 led to a remarkable increase in the HMW insoluble protein species of the two DPRs, making previously negligible differences with LIPO significant.

Using FRA to measure polyGP or polyPR levels in NSC34 cells treated with either MG132 or 3-MA, we found that VCP acts in different manner on the clearance of the various C9-DPRs. VCP promoted polyGP clearance through the UPS: the inhibition of UPS prevented VCP-mediated clearance, while the inhibition of autophagy did not impact the role of VCP. Conversely, polyPR clearance is mediated by both degradative pathways. Indeed, neither UPS nor autophagy inhibition prevented VCP activity in polyPR clearance. Thus, to verify this hypothesis, we contemporarily blocked both degradation systems using MG132 and NH_4_Cl (Fig. 5K). Through FRA, we confirmed VCP activity on polyPR and the shuttling of VCP on the alternative degradation mechanisms. Indeed, the simultaneous inhibition of UPS and autophagy prevents VCP activity. This suggests that the polyPR degradation induced by VCP is mediated by an alternative routing of the cargo to be eliminated between these two systems. These data underline that C9-DPR clearance can be regulated by VCP. In addition, data show that VCP has a multifaceted role, which becomes specific for each C9-DPR.

### SMER28-mediated VCP-dependent induction of the UPS decreases polyPR levels

**Fig. 6:**
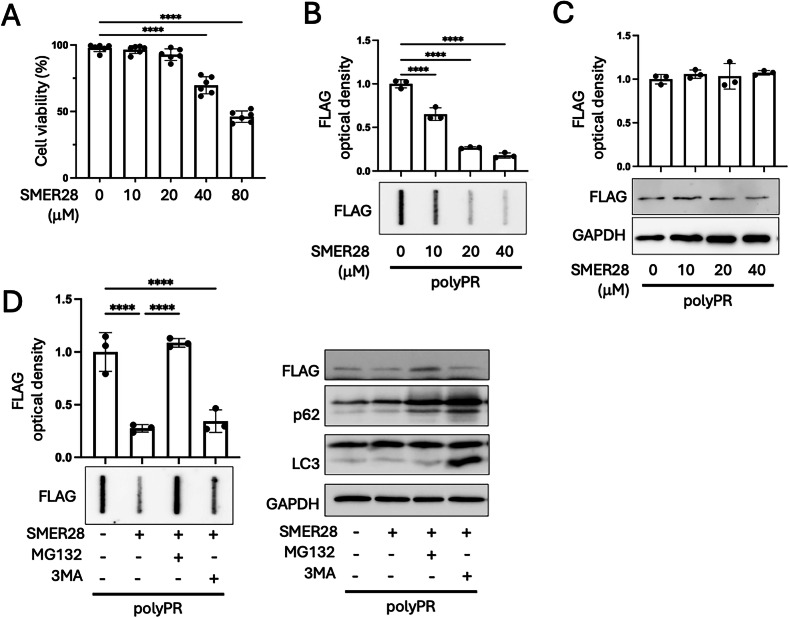
SMER28 induces polyPR clearance through UPS in NSC34 cells. **A** MTT cell viability assay was performed on NSC34 cells treated with SMER28 or DMSO for 48 h. (One-way ANOVA followed by Fisher’s LSD test; ****p < 0.0001). **B** Graph (upper inset) quantifying the FRA of PBS-protein extracts of NSC34 cells expressing polyPR and treated with SMER28 (lower inset). polyPR is visualized with an anti-FLAG antibody (One-way ANOVA followed by Fisher’s LSD test; ****p < 0.0001). **C** Graph (upper inset) quantifying WB on NSC34 cells expressing polyPR and treated with SMER28 (lower inset). polyPR is marked with an anti-FLAG antibody. GAPDH was used as a loading control. **D** Graph (left inset) quantifying FRA of PBS-protein extracts of NSC34 expressing polyPR and treated with SMER28 and proteasome or autophagic inhibitors, MG132/3-MA. In right inset a representative WB. polyPR is marked with anti-FLAG. Degradation systems markers p62 and LC3 are marked with anti-p62/LC3 antibodies. GAPDH is used as a loading control.

To further characterize the role of VCP, we used SMER28, a specific activator of VCP-mediated autophagic and proteasomal degradation [31]. This new approach activated a specific function of VCP, whereas our previous approaches based on VCP overexpression stimulated multiple pathways involving VCP. Thus, we examined the influence of SMER28 on polyPR expressed in NSC34 cells (Fig. 6). We selected polyPR since it is the most affected by VCP overexpression.

Initially, we evaluated the dose-dependent impact of SMER28 on NSC34 cell viability. Notably, SMER28 treatment at lower doses (up to 20 µM) did not significantly impact cell viability, whereas higher concentrations (40 and 80 µM) led to a dose-dependent loss of viability (Fig. 6A). We decided to use 20 µM for the following experiments. Thus, we analyzed the contribution of SMER28 to the clearance of the HMW insoluble polyPR fraction using FRA and total protein levels using WB (Fig. 6B, C). We found that SMER28 induced a dose-dependent decrease in polyPR aggregates (Fig. 6B) without affecting the total polyPR protein levels (Fig. 6C). Since SMER28 promotes the VCP-dependent activation of both proteasome and autophagy, we further investigated the preferential pathway involved in the VCP-mediated reduction of polyPR aggregates (Fig. 6D). Thus, NSC34 cells expressing polyPR were co-treated with SMER28 and either proteasome (MG132) or autophagy (3-MA) inhibitors (Fig. 6D). The data showed that proteasome blockage reverted the effect of SMER28, while autophagy inhibition did not significantly alter the contribution of SMER28. Thus, SMER28 modulation of VCP induces the clearance of polyPR aggregates through the proteasome.

### VCP modulation decreases C9-DPR-induced lysosomal damage

**Fig. 7:**
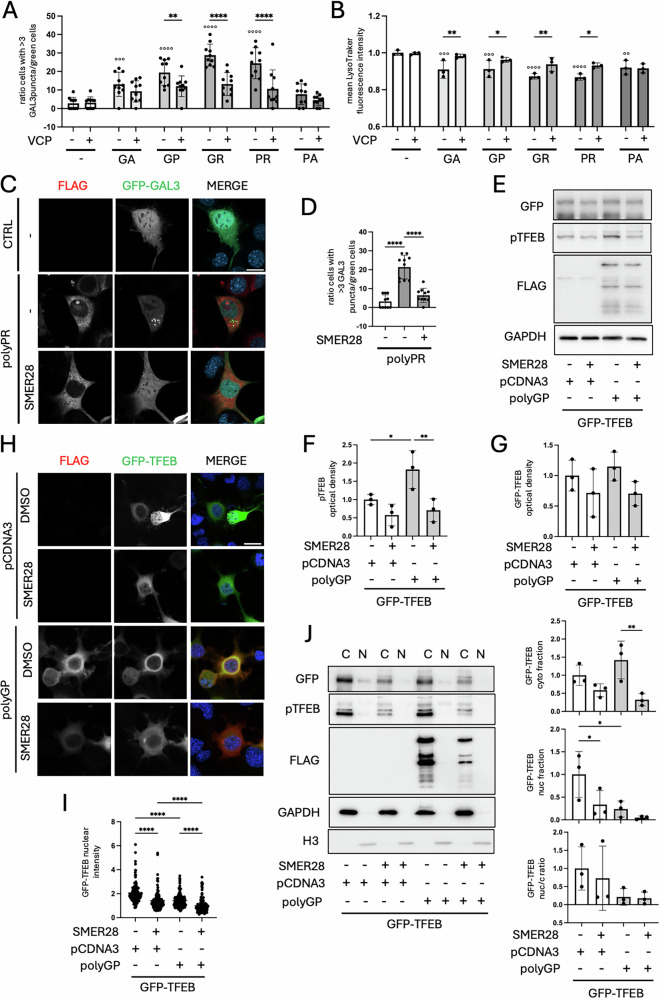
VCP modulation reverts DPR-induced lysosomal damage. **A**, **B** NSC34 cells expressing each DPR and VCP. **A** The bar graph represents the quantification of the percentage of cells with >3 GFP-LGALS3 puncta after transfection with pCDNA3 or DPRs; the fields were randomly selected and at least 100 cells for each sample were counted over 9 independent biological samples for each condition (*n* = 9) ± SD (One-way ANOVA followed by Fisher’s LSD test; **p < 0.01, ****p < 0.0001; °°°*p* < 0.001, °°°°*p* < 0.0001, vs CTRL). **B** The bar graph represents the quantification of the mean LysoTracker fluorescence intensity cytofluorimetric analysis on NSC34 cells labeled with LysoTracker Green. Mean fluorescence intensity was measured (*n* = 3) (One-way ANOVA followed by Fisher’s LSD test; *p < 0.05, °°*p* < 0.01; °°°*p* < 0.001, °°°°*p* < 0.0001, vs CTRL). **C** IF analysis (63x magnification) on NSC34 cells overexpressing polyPR and GFP-LGALS3. polyPR was stained with an anti-FLAG antibody (red), and nuclei were stained with DAPI (blue). Scale bar 10 µm. **D** The bar graph represents the quantification of the percentage of cells with >3 GFP-LGALS3 puncta after polyPR construct transfection and SMER28 or DMSO treatment; the fields were randomly selected, and at least 100 cells for each sample were counted over 9 independent biological samples for each condition (*n* = 9) ± SD (One-way ANOVA followed by Fisher’s LSD test; ****p < 0.0001). **E**–**L** NSC34 cells expressing GFP-TFEB and FLAG-polyGP/pCDNA3 and treated with SMER28 for 48 h. **E** WB of total protein. TFEB was marked with anti-GFP, and TFEB phosphorylated was marked with anti-pTFEB. Anti-FLAG shows polyGP expression. GAPDH was used as a loading control. **F**, **G** Quantification of pTFEB and GFP-TFEB (One-way ANOVA followed Fisher’s LSD test; *p < 0.05, **p < 0.01). **H** IF analysis (60x magnification). PolyGP was marked with an anti-FLAG antibody (red) and nuclei were stained with DAPI (blue). Scale bar 10 mm. **I** Quantification of GFP-TFEB nuclear intensity; the fields were randomly selected, and at least 100 cells were analyzed for each condition (one-way ANOVA with Kruskal-Wallis test; ****p < 0.0001). **J** Representative WB (left inset) of cytosol and nuclei fractions. To visualize polyPR, an anti-FLAG antibody was used. To visualize GFP-TFEB, anti-GFP or pTFEB antibodies were used. GAPDH and H3 were used as loading controls for cytoplasmic and nuclear fractions, respectively. Quantification of TFEB nuclear/cytoplasmic fraction or ratio is represented in the graphs (right inset) (One-way ANOVA followed by Fisher’s LSD test; **p < 0.01).

Since VCP modulation decreases C9-DPR levels, we evaluated whether this correlated with a rescue of C9-DPR pathological mechanisms. Hence, we analyzed the impact of VCP overexpression on lysosomal damage and functionality in NSC34 cells expressing C9-DPRs. We analyzed damaged lysosomes based on GAL3 redistribution and found that VCP decreased the number of GAL3 puncta associated with all C9-DPRs but polyGA and polyPA (Fig. 7A). Moreover, using LysoTracker, we confirmed an increase in functional lysosomes induced upon VCP overexpression in cells expressing polyGA, polyGP, polyGR, and polyPR (Fig. 7B).

Then, we activated VCP in NSC34 cells using the modulator SMER28 to confirm the role of VCP on polyPR, the most toxic DPR. PolyPR-induced lysosomal membrane damage was significantly reduced by SMER28 treatment (Fig. 7C). Furthermore, this was confirmed by GAL3 puncta quantification (Fig. 7D). We then studied whether VCP modulation could rescue TFEB nuclear level alterations triggered by the C9-DPRs. To this aim, we expressed GFP-TFEB in NSC34 cells in the presence of polyGP, the DPR mostly affecting TFEB localization, in the absence or presence of SMER28 (Fig. 7E–L). By analyzing the total phosphorylated TFEB (pTFEB, the inactive form) and TFEB protein levels (Fig. 7E–G), we observed that polyGP induced a significant increase in pTFEB levels in line with the cytoplasmic accumulation of pTFEB and the alteration of its nuclear levels. This abnormal induction of pTFEB was then rescued by SMER28 treatment. Notably, the quantification of total TFEB analyzed using the anti-GFP antibody did not show any significant differences, but a decreased trend in the presence of SMER28 treatment. Indeed, IF imaging on the same condition showed a significant decrease in the intensity of nuclear GFP-TFEB signal in the presence of SMER28 compared to the control and the untreated polyGP conditions (Fig. 7H, I). To better understand the SMER28 impact on TFEB, we performed a nuclear/cytoplasmic fractionation. We detected a decrease in both cytoplasmic and nuclear levels of GFP-TFEB in the presence of SMER28 treatment, which resulted in a significant increase in the cytoplasmic fraction in the presence of polyGP, and in the nuclear fraction in the absence of polyGP. In the other conditions tested, SMER28 still showed a decrease of TFEB in the nuclear/cytoplasmic fraction, although it did not result significant (Fig. 7J).

Together, these data show that VCP biological and chemical modulation positively impacts C9-DPRs toxicity by rescuing lysosome alterations. However, how and to what extent VCP modulation via SMER28 treatment affects TFEB mislocalization still needs further investigation.

### SMER28 treatment rescues pathological features in iPSC-MNs harboring C9orf72 mutation

**Fig. 8:**
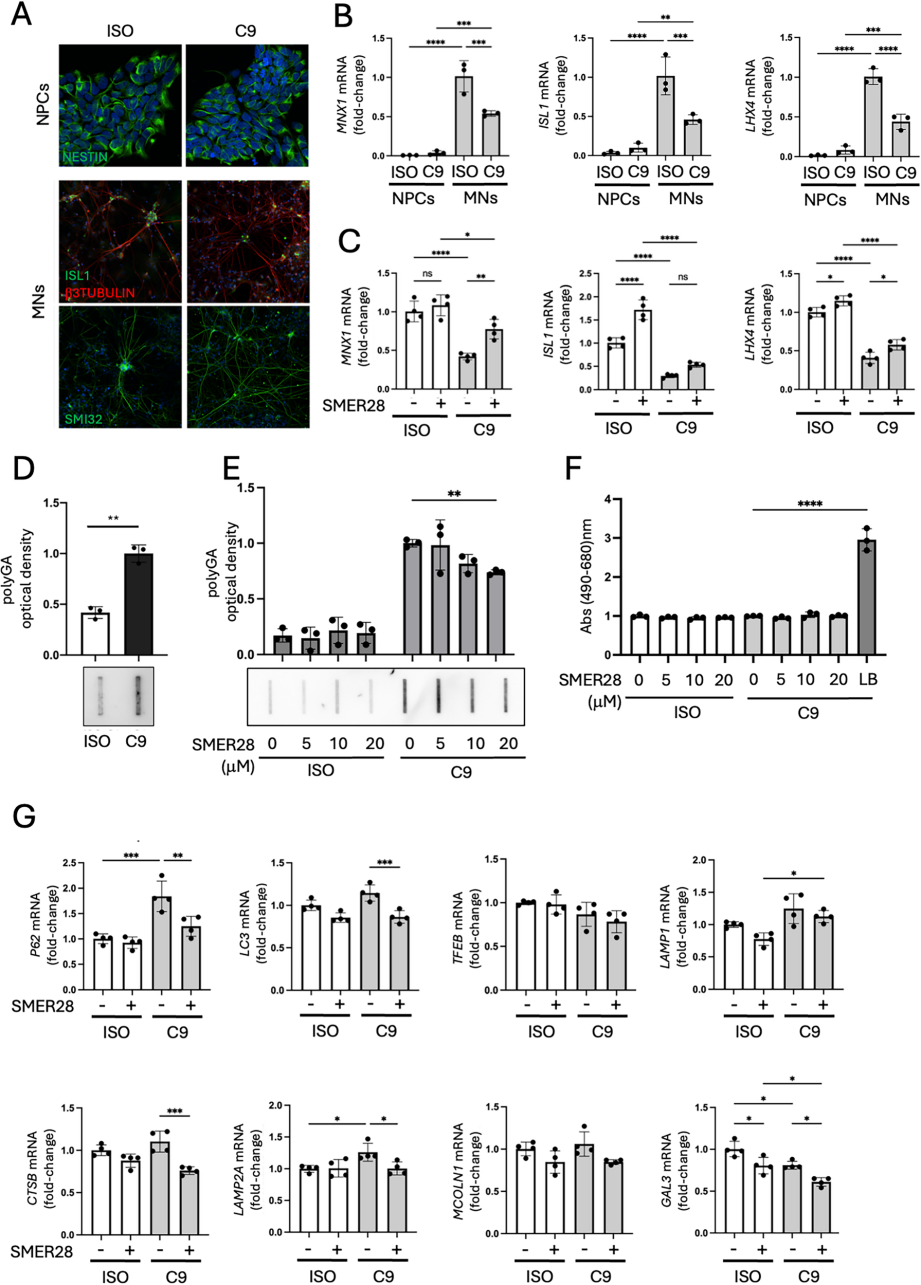
SMER28 reduces polyGA levels in iPSC-MNs. **A** IF analysis (10x magnification) on neural progenitor stem cells (NPCs) and motorneurons (MNs) differentiated from M2-C9 and its isogenic (ISO) corrected cell line (left inset). NPCs were stained with the NPC marker Nestin (green). MNs were stained with MN markers ISL1/SMI32 (green) and β3tubulin (red). Nuclei were stained with DAPI (blue). **B** RT-qPCR on C9-MNs/NPCs and ISO-MNs/NPCs for *MNX1*, *ISL1*, and *LHX4*, MNs markers, mRNA normalized with *RPLP0* mRNA levels. Data are means SD of 3 independent samples. **C** RT-qPCR on C9/ISO-MNs treated with SMER28. MNs markers mRNA normalized with *RPLP0* mRNA levels. Data are means SD of 4 independent samples (one-way ANOVA with Fisher’s LSD test; **p < 0.01; ***p < 0.001; ****p < 0.0001). **D** Bar graph (upper inset) of Immunoblot analysis on M2–MNs (lower inset). Anti-polyGA antibody marks polyGA protein levels (unpaired Student's t-test; **p < 0.01). **E** Bar graph (upper inset) of Immunoblot analysis on M2-MNs treated with different concentrations of SMER28 (lower inset). Anti-polyGA antibody marks polyGA protein levels (one-way ANOVA with Fisher’s LSD test; **p < 0.01). **F** LDH assay on M2-MNs treated with different concentrations of SMER28. Lysis buffer (LB) was used to evaluate the maximum LDH release (One-way ANOVA followed by Fisher’s LSD test; ****p < 0.0001). **G** RT-qPCR on M2-C9 and ISO MNs treated with SMER28 for *p62*, *LC3*, *TFEB*, *LAMP1*, *CTSB*, *LAMP2A*, *MCOLN1*, and *GAL3* mRNA normalized with Rplp0 mRNA levels. Data are means SD of 4 independent samples (one-way ANOVA with Fisher’s LSD test; *p < 0.05, **p < 0.01, ***p < 0.001).

Once the contribution of VCP in the rescue of C9-DPRs in NSC34 cells was proven, we further investigated VCP influence on C9-DPR clearance by translating our observations into a more sophisticated cell model. For this purpose, we utilized MNs obtained from iPSCs derived from a C9-ALS patient, which currently represents the best cell model for this pathology and could also be used for a proof of principle for developing a therapeutic molecule. Initially, we generated the model by differentiating C9-iPSCs (M211R2_C9, or M2) and the isogenic cell line (ISO) following a small molecule-based approach (described in [36]). Differentiated MNs was obtained via an intermediate step in which we generated neural progenitor cells (NPCs) positive for Nestin, an intermediate filament marker of neural progenitors (Fig. 8A). At the end of the differentiation process, we obtained a mixed neuronal population which presented cells positive to Islet1 (ISL1), Motor Neuron and Pancreas Homeobox 1 (MNX1) and LIM Homeobox 4 (LHX4), transcription factors expressed in MNs; β3tubulin; an early marker of differentiation of both central and peripheral neurons SMI-32; a neurofilament protein that marks pyramidal neurons (Fig. 8A, B). We then quantified DPR levels using the immunoblot assay in MNs. Using an antibody against polyGA, the most abundant DPR, we detected its presence in the C9 cell line whereas isogenic cell line presents a faint signal considerable background (Fig. 8D).

We then evaluated if the pharmacological activation of VCP enhanced the polyGA clearance by treating MNs with different concentrations of SMER28 for 48 h (Fig. 8E). Immunoblot analysis revealed that SMER28 significantly decreased polyGA levels without influencing MNs viability (Fig. 8D, F). The effect of SMER28 was further confirmed by treating MNs obtained from a different C9-iPSCs line (CS52iALS), in which we confirmed the reduction in polyGA levels after SMER28 treatment (Supplementary Fig. 4A). Unexpectedly, we did not find any alteration of cell viability in C9-MNs in comparison to ISO-MNs (Fig. 8F) Therefore, to assess if SMER28 could ameliorate C9-MNs sensitivity to stress, we treated MNs with different concentration of glutamate and with or without SMER28. Then, after 48 h, we evaluated cell toxicity using the LDH assay (Supplementary Fig. 4C). Surprisingly, no significant changes in cell viability were observed under any of the conditions tested.

We also determined the impact of SMER28 on MNs differentiation by quantifying the expression of MN markers (Fig. 8C). The analyses showed that, in basal conditions, C9-MNs presented lower levels of *MNX1*, *ISL1*, and *LHX4* in comparison with its isogenic cell line, while SMER28 treatment significantly increased the levels of *MNX1*, *ISL1* expression in C9-MNs. A positive trend was also visible in *LHX4* expression levels. Collectively, these data strongly suggest that SMER28 rescues the differentiation defects characterizing C9-MNs.

Moreover, we evaluated the expression of autophagy-related genes in C9-MNs and ISO-MNs (Fig. 8G). We observed that compared to ISO-MNs, C9-MNs presented a significant increase in the expression of *p62*, *LAMP2A*, and a decrease in the levels of *GAL3*. We also note a trend of increase in *LC3*, *LAMP1*, and *CTSB*, which did not reach a statistical value, while *TFEB* expression remained unchanged. A similar expression pattern was confirmed in the CS52iALS line (Supplementary Fig. 4B). In this context, SMER28 treatment resulted in a reduced expression of all the abnormally elevated genes considered, rescuing the C9-associated alterations.

Finally, given that C9-MNs are known to be sensitive to lysosomal alterations [37], we assessed the presence of damaged lysosomes positive for GAL3 in differentiated iPSCs. We observed an increased number of GAL3-positive puncta in C9-differentiated cells, which was rescued by SMER28 treatment (Supplementary Fig. 4D). However, additional experiments are required to better characterize lysosomal alterations in this model.

Overall, SMER28's impact on iPSC-MNs emphasizes the importance of VCP-mediated clearance in the turnover of C9-DPRs and suggests it as a potential target to rescue C9 pathology.

## Discussion

The *C9* mutation consists of an abnormally expanded hexanucleotide (G_4_C_2_)_n_ repeat, which is associated with ALS and FTD, two devastating and disabling diseases with no cure. The pathological mechanisms linked to the *C9* mutation involve effects at both mRNA and protein levels, leading to C9 haploinsufficiency, RNA foci formation, and aberrant translation and aggregation of C9-DPRs. Given their distinct toxic properties, the five C9-DPRs tend to aggregate and damage cells in different ways, such as by sequestering essential proteins for cell homeostasis and/or by inducing alterations in specific organelles, such as lysosomes, which can be fatal to cells [38].

Here, by using two neuronal models, we outlined a novel strategy to promote C9-DPR clearance and counteract pathological mechanisms believed to result from C9-DPRs aggregation and its detrimental impact on cellular homeostasis.

We first showed that immortalized motoneuronal NSC34 cells expressing the different C9-DPRs exhibit disruption of the ALP, resulting in increased lysosome membrane permeabilization, altered lysosome pH and activity, and lysosome enlargement. Specifically, the expression of all C9-DPRs leads to an alteration in lysosomal morphology and size, as observed with TEM. As previously described, each C9-DPR impacts lysosomal function in distinctive ways, collectively deteriorating ALP. These morphological alterations are accompanied by a redistribution of GAL3, a marker of lysosomal damage, and modification of lysosomal pH, visualized with LysoTracker. Also, while all C9-DPRs contributed to lysosomal damage to a different extent, the effect was more pronounced for the two most toxic C9-DPRs, polyPR and polyGR. The observed lysosomal impairment in this model is in line with findings in the literature. Beckers and colleagues reported that C9-iPSC-MNs presented lysosomes altered in their morphology, size, and activity [37]. These data were only partially recapitulated in *C9*-knockout iPSC-MNs, underlining the pathological relevance of the toxicity induced by C9-DPRs. C9 fly models previously examined by Cunningham and colleagues, too, presented ALP disruption, specifically in terms of lysosome dysfunction and increased number of MLBs, a marker of dysfunctional lysosomes and autophagy [19, 39].

We and others have previously shown that important alterations in lysosomes, such as lysosomal damage and accumulation of altered lysosomes, activate the transcription factors TFEB and TFE3. Their activation promotes lysosome degradation and biogenesis, which prevents damaged lysosome accumulation and the resulting cell toxicity [25]. Thus, we investigated TFEB/TFE3 activation in our model, but we failed to observe a change in the expression of autophagy-related genes. However, we found that C9-DPRs alter TFEB/TFE3 distribution and decrease their nuclear levels. Again, each C9-DPR produces a distinct effect: polyGP and polyPA significantly decrease TFEB and TFE3 nuclear/cytoplasmic ratio; polyPR impacts mainly TFE3 nuclear/cytoplasmic ratio; polyGP, polyPR, and polyGR induce a notable perinuclear accumulation of TFEB. In line with our findings, Cunningham and colleagues observed impaired nuclear import of Mitf, the TFEB fly homolog [19]. While the exact mechanism preventing Mitf/TFEB translocation remains unknown, increasing Mitf/TFEB levels or inhibiting its cytoplasmic export has been shown to improve C9-linked neurodegenerative phenotype [16]. C9-DPRs have been widely described to generally alter nuclear import/export. PolyGA cytoplasmic aggregation correlated with the disruption of the importin-α/β-dependent nuclear import of TDP-43. Overexpression of importin-α/β but also of the nuclear pore components NUP54 and NUP62 restores proper TDP-43 nuclear levels [40]. Thus, it is likely that the shuttling system of TFEB/TFE3 or the direct alteration of the nuclear pores could disrupt proper TFEB/TFE3 localization. Further studies are needed to better understand these mechanisms.

TFEB/TFE3 activation is essential for lysosomal biogenesis and autophagy regulation. Consequently, the disruption of their nuclear localization exacerbates lysosomal dysfunction, preventing the restoration of a functional lysosome pool.

A key modulator of the clearance of damaged lysosomes and protein aggregates is VCP. VCP regulates lysophagy by removing and promoting the degradation of specific lysosome membrane proteins. Their removal is needed for damaged lysosome engulfment in the autophagosome [41]. The critical role of VCP in lysophagy is further shown by the fact that mutations in *VCP* result in impaired lysophagy and accumulation of damaged lysosomes [25, 42]. In C9 models, as previously mentioned, VCP was shown to directly interact with polyGA, suggesting its involvement in the clearance of C9-DPRs [32]. However, we could not prove a co-localization between VCP and any of the five C9-DPRs in NSC34. Nevertheless, in regard to VCP and C9-DPRs interaction, we observed that: polyPR nuclear aggregates promote VCP re-localization into the nucleus; polyGP directly interacts with VCP; VCP depletion triggers C9-DPRs toxicity and cell death. Overall, our data show an essential role of VCP, either directly or indirectly, in C9-DPR clearance, and/or its role could be beneficial in mitigating C9-related dysfunctional ALP. Therefore, we modulated VCP to better define its beneficial effects on C9 models.

Our results demonstrated that VCP plays a crucial role in reducing the accumulation of C9-DPRs. Specifically, VCP overexpression led to a decrease in the HMW insoluble species of all C9-DPRs, except for polyPA. We then further investigated the mechanism involved in VCP-mediated clearance of C9-DPRs, alternatively inhibiting the UPS or autophagy, as VCP is implicated in both degradative pathways [43, 44]. We observed that the elimination of C9-DPRs can be alternatively facilitated through either the UPS or autophagy. However, the precise mechanism governing the switch between these degradation pathways remains to be elucidated. This variability may be influenced by the distinct biochemical properties of each C9-DPR, such as solubility, charge, and subcellular localization, as well as by the high functional plasticity of VCP. A particularly interesting mechanistic finding is that VCP directly interacts with polyGP and promotes its degradation via the UPS. In contrast, we found no evidence of a direct interaction between VCP and polyPR, and VCP-mediated polyPR clearance appears to involve a more flexible mechanism. Indeed, VCP overexpression reduced polyPR levels in a manner that was not exclusively dependent on either the UPS or autophagy. In fact, inhibition of VCP-mediated polyPR clearance was observed only when both pathways were simultaneously blocked, indicating shuttling between the two degradative systems mediated by VCP. More experiments need to be performed to define the conditions that address the mechanism in a specific pathway.

In addition, we observed that VCP modulation could also revert C9-DPRs detrimental impact on lysosomes. In cells expressing C9-DPRs, VCP overexpression positively influenced lysosomal function, as evidenced by decreased lysosomal damage and restored lysosomal pH. The re-establishment of functional lysosomes may result from a decrease in C9-DPR aggregates but also from a direct increase in lysophagy.

As VCP is implicated in many pathways of the PQC, its overexpression could result in the activation of various processes. To specifically trigger VCP activity in autophagy and UPS degradation, we took advantage of a pharmacological approach using SMER28. This compound has been previously tested in other NDs. For instance, it showed a positive effect on toxic aggregates reduction in models of Huntington disease and spinocerebellar ataxias [31]. Here, SMER28 treatment further confirmed the role of VCP in polyPR clearance through a UPS-dependent mechanism and its role in decreasing the lysosomal damage induced by polyPR. We also evaluated SMER28's contribution in re-establishing TFEB activation and localization. In this regard, we found a decrease in the polyGP toxicity with a rescue in pTFEB levels; however, SMER28 decreased TFEB protein stability in general, a phenomenon possibly related to an increased autophagic activation induced by SMER28 already previously demonstrated in other studies [31].

After defining the VCP mechanism of rescue in C9-DPRs pathology in an immortalized model, we evaluated the VCP contribution in iPSC-MNs harboring the *C9* mutation. Indeed, NSC34 cells are highly valuable, permitting to deeply understand mechanisms in a relatively short time, but have limitations linked to the immortalization and the genetic background, which differs from that of a C9-patient. Since polyGA levels are well-characterized in neuronal models derived from patients, we used polyGA as a readout for DPR levels. Unfortunately, polyGR and polyPR, which are known to be more toxic, cannot be easily quantified in these models. Notably, iPSC-MNs treatment with SMER28 resulted in a significant decrease in polyGA levels. We also proved that SMER28 increases differentiation of C9-MNs and rescues the altered expression of autophagy-related genes. Still, the exact mechanisms that correlate *C9* mutation to an increased expression of different autophagy-related genes have to be defined. This phenomenon can be attributed to a defense mechanism activated as a consequence of an alteration of the PQC system. Overall, these data highlight the translational relevance of our findings.

In conclusion, our study underlines the pivotal role of VCP in regulating proteostasis and lysosomal function in models of ALS and FTD associated with the *C9* mutation. These findings suggest that VCP modulation is a promising therapeutic strategy to mitigate or prevent the toxic effects of C9-DPRs and restore cellular homeostasis.

## Material and Methods

### Chemicals

The following chemicals were used:

MG132 (Sigma-Aldrich, C2211) 10 μM for 16 h for proteasome inhibition;

3-MA (Sigma-Aldrich, M9281) 10 mM for 48 h for autophagy inhibition;

NH_4_Cl (Euroclone, EMRO895009) 4 mM for 16 h for autophagy inhibition;

SMER28 (Cayman, CAY-17768) for 48 h (see figure legends for concentration).

### Cell cultures and transfection

Mouse motoneuron-like hybrid cell (NSC34) line [45, 46] was cultured in DMEM high-glucose medium (EuroClone, ECB7501L) supplemented with 5% fetal bovine serum (Sigma-Aldrich, F7524), 1 mM L-glutamine (EuroClone, ECB3004D), and penicillin-streptomycin solution (EuroClone, ECB3001D), and grown at 37 °C in 5% CO_2_. NSC34 cells were plated at a density of 80,000 cells/ml and were transfected with 1 μg/ml of DNA plasmid using Lipofectamine 3000^®^ (ThermoFisher Scientific, L3000015) or with 20-40pmol/ml of siRNA.

The plasmids used for the transfection are:

FLAG-tagged plasmids coding for scrambled-GA, scrambled-GP, scrambled-GR, scrambled-PR, scrambled-PR (100 repeats), kindly provided by Professor Daisuke Ito (Keio University School of Medicine, Tokyo, Japan) (Yamakawa et al., 2015). These plasmids were optimized to encode for the single DPRs.

HIS-tagged plasmids coding for VCP. p6xHIS-VCP were obtained by excising the FLAG tag from

pFLAG-VCPs with HindIII/EsprI and inserting an in-frame 6xHIS sequence.

pEGFP-LGALS3 was obtained from Prof. M. A. Jäättelä (Danish Cancer Society Research Center, Copenhagen, Denmark).

pTFEB-eGFP-N1 plasmid was kindly provided by Prof. A. Ballabio (Telethon Institute of Genetics and Medicine (TIGEM), Dulbecco Telethon Institute, Federico II University, Naples)

pCDNA3 was obtained from Life Technologies (V790-20). It was used as a transfection control.

pEGFPN1 was obtained from Clontech-Lab (U55762) and was used to evaluate transfection efficiency by fluorescent microscopy.

The siRNAs used were purchased from Dharmacon. The sequences are the following:

non-targeting (NT) siRNA Sense: 5ʹ-UAGCGACUAAACACAUCAAUU-3ʹ; Antisense: 5ʹ-UUGA UGUGUUUAGUCGCUAUU-3ʹ;

*Vcp* siRNA: Sense: 5’-GUUCAAAGUUGUAGAGACAUU-3’; Antisense: 5’-UGUCUCUACAACUUUGAACUU-3’

CS52iALS-C9n6 and CS52iALS-C9n6.ISOC3 iPSC lines were bought from Cedars-Sinai Medical Center iPSC Core Facility, Los Angeles, USA. M211R2_C9 and M211R2_ISO iPSC lines were kindly given by Dr Bhuvaneish Selvaraj and Prof Siddharthan Chandran (The University of Edinburgh, Edinburgh). Cells were cultured on Matrigel (Corning, 354277) and fed with E8 Flexi (ThermoFisher Scientific, A1517001). Every 5–6 days, cells were split using Accutase (Merck, A6964), and on the first day, E8 was supplemented with ROCK-I (5 μM; Selleckchem, S1049).

iPSCs were differentiated into small molecule neural progenitor cells (smNPCs) following the already published protocol [36]. In brief, embryo bodies were derived from iPSCs and were maintained in a medium (N2B27 medium) composed of DMEM HAM’S F-12 (Euroclone, ECM0090L), Neurobasal medium (ThermoFisher Scientific, 21103049), N-2™ supplement (ThermoFisher Scientific, 17502048), B-27™ supplement (ThermoFisher Scientific, 17504044), and antibiotics. Cells were treated with CHIR99021, SB431542, LDN 193189, SAG, and Ascorbic Acid (AA). After 6 days, embryo bodies were dissociated and plated on Matrigel (hESC-qualified Matrix; Corning®, 354277) in a medium composed of N2B27 with CHIR99021, AA, and SAG. smNPCs were cultured for 6–7 passages before starting differentiation to motor neurons (MNs). MNs differentiation was achieved by plating smNPCs at 150,000 cells/ml in Patterning medium (N2B27 medium supplemented with AA, CHIR99021, BDNF, GDNF, and retinoic acid). After 6 days, cells were changed to Maturation medium (N2B27 supplemented with dbCAMP, AA, GDNF, BDNF, TGFΒ3, and Activin A). After two days, the cells were replated at 350,000 cells/ml and maintained up to 10 days.

### RT-qPCR

RT-qPCR performed on NSC34: cells were collected, and total RNA was extracted using Tri-Reagent (Sigma- Aldrich, T9424) following the manufacturer’s protocol; 1 μg per sample was treated with DNase and reverse transcribed using the High-Capacity cDNA Reverse Transcription Kit (ThermoFisher-Scientific, 4368814).

RT-qPCR was performed using the CFX96 Real-Time System (Bio-Rad Laboratories), the iTaq SYBR Green Supermix (Bio-Rad Laboratories, 1725124), and with a final concentration of 500 nM of primers. Data were normalized using *Rplp0*. The experiments were performed with 4 independent samples (*n* = 4). All primers used are listed in Supplementary Table 2.

RT-qPCR performed on iPSC-MNs: cells were collected, and the total RNA was extracted and processed with DNase using Quick-RNA MicroPrep Kit (Zymo Research, R1051) following the manufacturer’s protocol. RNA was quantified using a NanoDrop 2000 (ThermoFisher Scientific, ND-2000), and 0.5 μg per sample was reverse transcribed using the High-Capacity cDNA Reverse Transcription Kit (ThermoFisher Scientific, 4368814). RT-qPCR was performed as described for NSC34 cells. All primers used are listed in Supplementary Table 2.

### Filter retardation assay and western blot analysis

To evaluate DPRs' HMW insoluble species, FRA was used. To evaluate total protein levels, WB was used. NSC34 cells were collected and centrifuged at 100 *g* for 5 min at 4 °C. Cell pellets were resuspended in phosphate-buffered saline (PBS; Sigma-Aldrich, P4417) supplemented with Protease Inhibitor Cocktail (Sigma-Aldrich, P8340), and an ultrasonic homogenization was performed. The protein extract concentration was quantified with the bicinchoninic acid method using a Quantum Protein Assay Kit (Cyanagen PRTD1,0500).

Samples (6 μg) were filtered on a 0.22 μm cellulose acetate membrane (Whatman GE Healthcare, GEH10404180) using a Bio-Dot SF Microfiltration Apparatus (Bio-Rad Laboratories, 1703938).

Protein extracts (20 μg) were loaded on 12% SDS–polyacrylamide gel electrophoresis. Samples were then electro-transferred to a nitrocellulose membrane (Amersham™ Protran®, GEH10600003) using a trans-Blot apparatus (Mini Trans-Blot Cell; Bio-Rad Laboratories).

Membranes were then blocked with 5% non-fat dried milk in Tris-buffered saline with 0.01% Tween (TBS-T) and subsequently incubated with the primary antibody overnight (o/n) at 4°C and the secondary antibody for 1 h at room temperature (RT). Signals were revealed by chemiluminescence detection kit reagents ECL Antares (Cyanagen, XLS142) or Westar hypernova (Cyanagen, XLS149). All antibodies used are listed in Supplementary Table 1.

### Immunofluorescence analysis

For immunofluorescence (IF) on NSC34 cells, we followed the following protocol: cells were fixed with 4% paraformaldehyde solution, permeabilized for 15 min using 0.2% Triton X-100 in PBS, and incubated for 1 h at RT in blocking solution. Cells were incubated o/n at 4 °C with primary antibody and 1 h at RT with secondary antibody. Nuclei were stained with DAPI (1:10,000 in PBS). Coverslips were mounted using Mowiol^®^ 4-88 (Merck-Millipore, 475904). Images were acquired with a Meta system confocal microscope (Zeiss). All antibodies used are listed in Supplementary table 1.

For IF analysis on smNPCs and MNs, we followed the following protocol: cells were fixed as described for NSC34 cells, then permeabilized and blocked for 45 min in PBS added with Triton X100 (0,1%), BSA (1%), and FBS (10%) at RT. To mark antigens, cells were incubated o/n at 4°C with primary antibody and 1 h at RT with secondary antibody. Nuclei were stained with DAPI (1:10,000 in PBS). Coverslips were mounted using Mowiol^®^ 4-88 (Merck-Millipore, 475904). Images were acquired with a Meta-system confocal microscope (Zeiss). All antibodies used are listed in Supplementary table 1.

### Galectin puncta assay

NSC34 cells with >3 GAL3 puncta were manually quantified in 3 randomly selected fields per sample (*n* = 9) condition using an eyepiece PL20 with graticules (10 mm in 100-grid divisions) as described in Rusmini et al. [34]. For each condition, 3 samples were analyzed. Cells expressing GFP and cells with >3 GFP-GAL3 puncta were counted in the same field. Their ratio was quantified in each field, and statistical analysis was performed.

### LysoTracker analysis

Relative lysosomal acidification status was measured in NSC34 incubated with 100 nM of lysosomotropic probe LysoTracker Green DND-26 (ThermoFisher Scientific, L7526) for 30 min. Then, cells were collected, resuspended in 4% FBS in PBS, and analyzed with a NovoCyte flow-cytometer (Acea Biosciences, Inc.). The mean LysoTracker fluorescence intensity was recorded from 50,000 cells for each sample (*n* = 4).

### TEM analysis

NSC34 cells were seeded at 180,000 cells/well in a 2-well Nunc® Lab-Tek®Chamber Slide™ system (Nunc, C6682). Transfected cells with different DPRs were fixed using 2.5% glutaraldehyde (Sigma-Aldrich, G7776) in 0.1 M sodium cacodylate buffer (Sigma-Aldrich, C0250), pH 7.4, for 1 h at room temperature. The cells were postfixed in osmium tetroxide (Electron Microscopy Science, 19100) for 2 h, and 1% uranyl acetate (SERVA Electrophoresis, 77870) for 1 h. Subsequently, samples were dehydrated through a graded ethanol series and flat-embedded in resin EMBED-812 (Electron Microscopy Science, 14120) for 24 h at 60 °C. Ultrathin sections (50 nm) were cut parallel to the substrate, stained with 5% uranyl acetate in 50% ethanol, and observed with a Hitachi TEM HT7800 microscope operating at 100Kv (Hitachi, Tokyo, Japan). Digital images were taken with a Megaview 3 camera. Analysis of morphologically identified single-membrane lysosomes (Lys) diameters was assessed in 10 cells for each DPR. The diameter of these structures was measured with the Radius 2.0 software package (EMSIS, Muenster, Germany) and plotted as scatter dot plots.

### MTT assay

The 3-(4,5-dimethyl-2-thiazolyl)-2,5 diphenyl-2H-tetrazolium bromide (MTT; Sigma-Aldrich, M2128)-based cell proliferation assay (MTT assay) was performed on NSC34 cells after 48 hours of treatment with different concentrations of SMER28 in MW24 plates seeded at 80,000 cells/ml. The culture medium was removed, and cells were incubated with MTT solution (1.5 mg/ml) at 37 °C for 30 min; then, isopropanol was added to block the reaction and solubilize the precipitates. Absorbance of each well was measured at 570 nm wavelength using an Enspire® Multimode plate reader (PerkinElmer, Waltham, MA, USA).

### LDH assay

To analyze the toxicity in NSC34, the CyQUANT™ LDH Cytotoxicity Assay Kit (Thermo Fisher Scientific, C20301) was used. Cells were analyzed 48 h after transfection. As a positive control, the maximum LDH release was measured on cells incubated with a supplied lysis buffer for 45 min at 37 °C. The test was then performed on 50 μL of supernatant, following the manufacturer’s protocol. The absorbance was measured at 490 nm and 680 nm wavelengths, using an Enspire® Multimode Plate Reader (PerkinElmer, Inc., Waltham, MA, USA).

### Immunoblot assay

PolyGA levels were analyzed with an Immunoblot. The cells were collected as described for NSC34 cells. 6 μg of total proteins were filtered through a 0.22 μm nitrocellulose membrane (Whatman GE Healthcare, GEH10404180) using a Bio-Dot SF Microfiltration Apparatus (Bio-Rad Laboratories, 1703938). Membranes were analyzed as described for the FRA. Membranes were incubated o/n at 4°C with the anti-polyGA primary antibody (see Supplementary table 1).

### Statistical analysis

The data are presented as mean ± SD. An unpaired *t*-test was used when two groups were present. One-way analysis of variance (ANOVA) was used, followed by a post hoc test as described in the legends, in the presence of 3 or more groups. Two-way ANOVA was performed to compare the effect of 2 independent variables when 3 or more groups were present. *P* values < 0.05 were considered statistically significant. Gaussian distribution was assumed in the *n* = 3 analysis, and equal variance was confirmed by the Brown–Forsythe test. All the analyses with *n* > 3 were tested with the Shapiro-Wilk or D’Agostino-Pearson test to check normal distribution. When values did not follow a normal distribution, a nonparametric test was used (the Kruskal-Wallis test). All the analyses were undertaken with the PRISM (version 8.2.1) software.

All experiments presented were conducted using 3 biological replicates (*n* = 3). If more experiments were considered, it has been outlined in the legends. The experiments have been replicated at least 3 times in the laboratory. No samples have been excluded.

## Supplementary information

Supplementary Materials

UNCROPPED_REVISED WB and FRA

## Data Availability

Data supporting the findings of this study are available from the corresponding author upon request.
