# Supplementary material for: VCP modulation ameliorates pathological features in C9orf72 models
Source: Cell Death Dis. 2026 May 17;17(1):629. doi: 10.1038/s41419-026-08856-1 (PMC13346533; doi:10.1038/s41419-026-08856-1)
Supplement: Supplementary file 1 — Supplementary Materials [file 41419_2026_8856_MOESM1_ESM.pdf]

SUPPLEMENTARY INFORMATION

VCP modulation ameliorates pathological features in C9orf72 models.

Veronica Ferrari[1], Barbara Tedesco[1], Marta Cozzi[1], Paola Pramaggiore[1], Maria Cristina Gagliani[2], Rocio Magdalena[1], Laura Cornaggia[1], Elena Casarotto[1], Marta Chierichetti[1], Ali Mohamed[1], Maria Brodnanovà[1], Carmelo Milioto[1], Margherita Piccolella[1], Mariarita Galbiati[1], Valeria Crippa[1], Alessandro Provenzani[3], Katia Cortese[2], Paola Rusmini[1], Riccardo Maria Cristofani[1], Angelo Poletti[1]

Supplementary Figure 1.

A

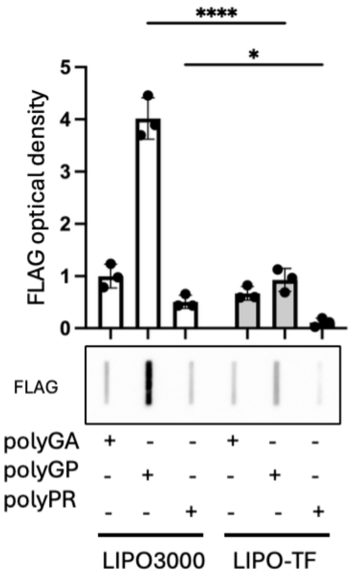

B

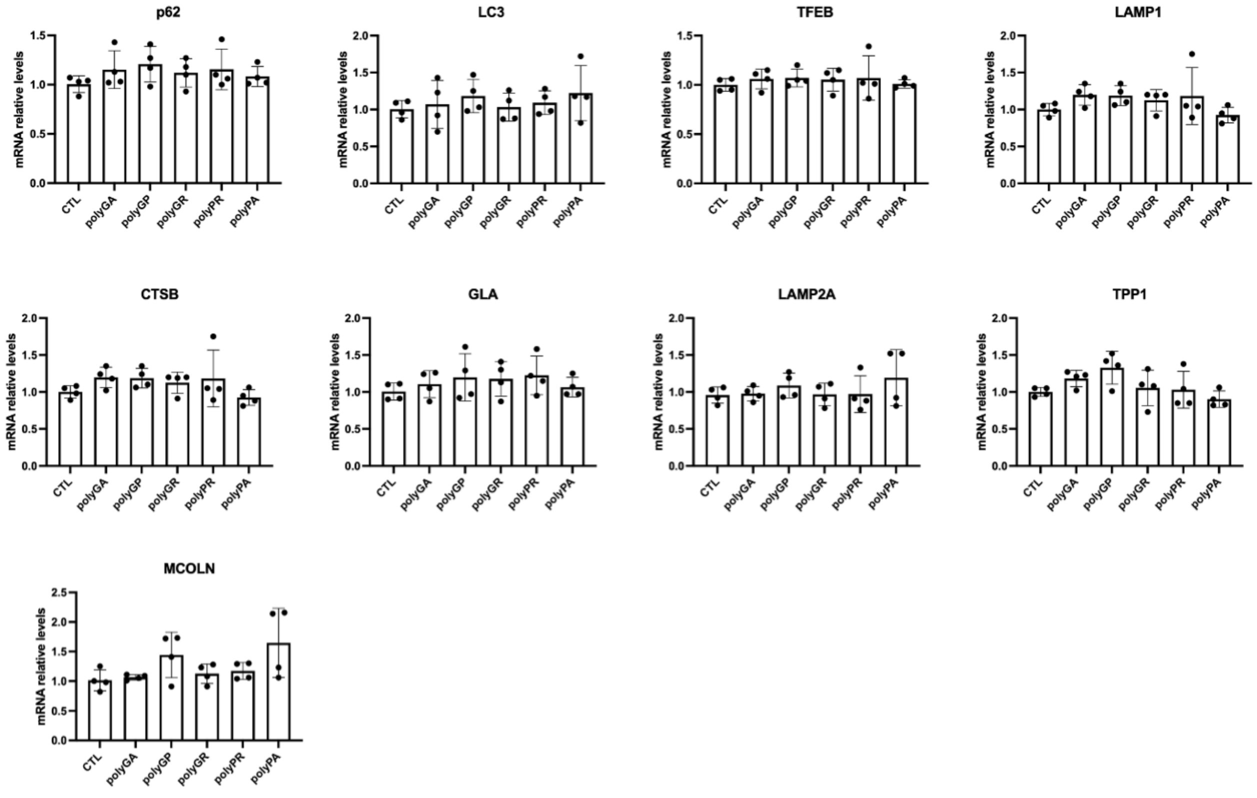

(A) NSC34 cells expressing polyGA, polyGP and polyPR transfected either with LIPO3000 or LIPO and transferrin. FRA analysis was performed on PBS-protein extracts. An anti-FLAG antibody was used to visualize DPRs. The bar graph quantifies FRA. (Two-way ANOVA followed by Fisher's LSD test; *$p<0.05$; ****$p<0.0001$). We compared the effect of lipofectamine (LIPO) and LIPO3000 on polyGA, polyGP, and polyPR accumulation. Transfection of plasmids encoding for polyGP and polyPR with LIPO3000 resulted in

an increased accumulation of these DPRs in FRA compared to LIPO, probably due to a more efficient transfection. PolyGA levels remained unchanged, which is in line with its low tendency to aggregate. (B) RT-qPCR on NSC34 cells expressing C9-DPRs for p62, LC3, GLA, TFEB, LAMP1, CTSB, LAMP2A, MCOLN and TPP1 mRNA normalized with Rplp0 mRNA levels. Data are means SD of 4 independent samples.

Supplementary Figure 2.

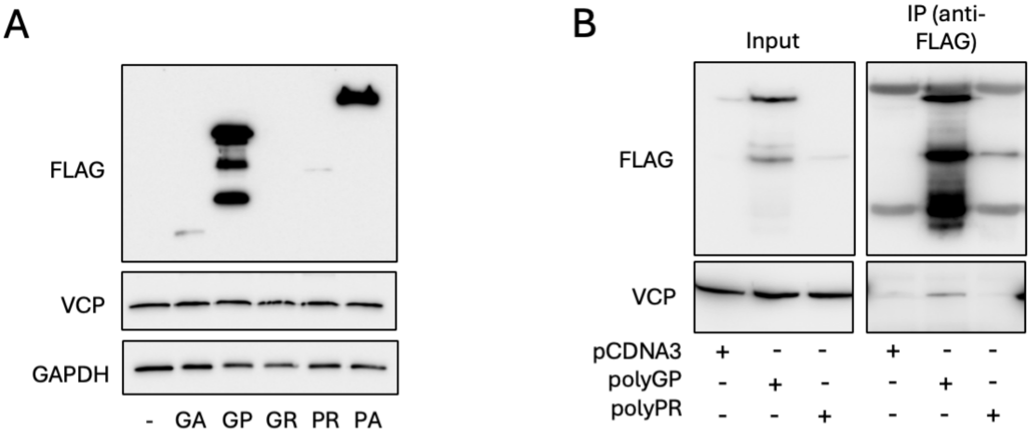

(A) NSC34 cells expressing polyGA, polyGP, polyGR, polyPR, polyPA. Representative WB of SDS-protein extracts. Each DPR was marked with an anti-FLAG antibody. Endogenous VCP was visualized with an anti-VCP antibody. GAPDH used as loading control. (B) Immunoprecipitation of NSC34 expressing polyGP/polyPR and pCDNA3 using an anti-FLAG antibody. FLAG antibody was used to detect polyGP and polyPR. Anti-VCP antibody was used to detect endogenous VCP.

Supplementary Figure 3.

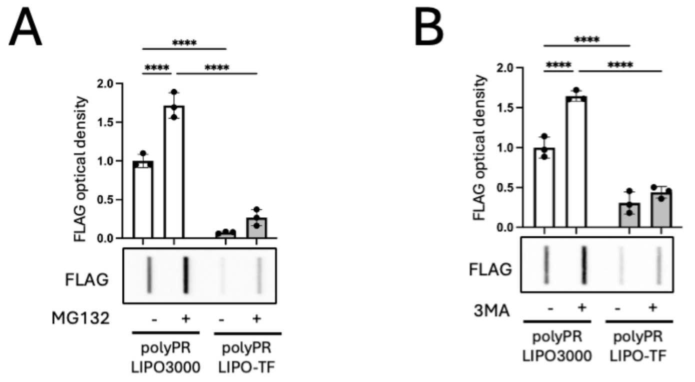

(A, B) NSC34 cells expressing polyPR transfected with LIPO3000 or LIPO and transferrin and treated with MG132 (A) or 3-MA (B). FRA analysis was performed on PBS-protein extracts. An anti-FLAG antibody was used to visualize polyPR. The bar graph quantifies FRA (Two-way ANOVA followed by Fisher's LSD test; ****p<0.0001).

Supplementary Figure 4.

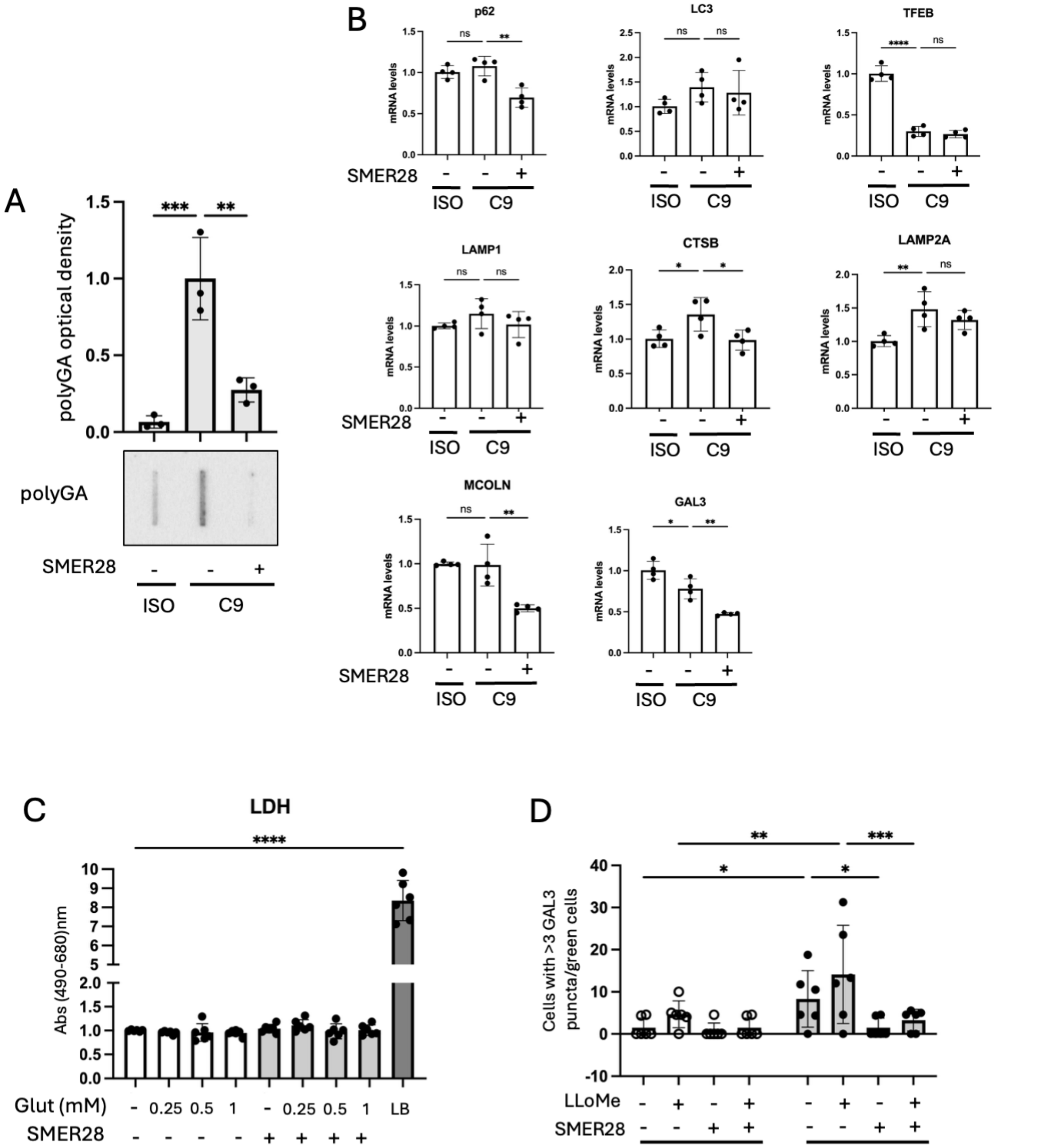

(A) Immunoblot with anti-polyGA antibody on CS52iALS-C9n6 and isogenic MNs (lower inset). Graphic quantification of polyGA in upper inset (one-way ANOVA with Fisher's LSD test; *p < 0.05, **p < 0.01). (B) RT-qPCR on CS52iALS-C9n6 and isogenic MNs treated with SMER28 for p62, LC3, HSPB8, TFEB, LAMP1, CTSB, LAMP2A, MCLN1 and GAL3 mRNA normalized with Rplp0 mRNA levels. Data are means SD of 4 independent samples (one-way ANOVA with Fisher's LSD test; *p < 0.05, **p < 0.01, ***p < 0.001). (C) LDH assay on M2-MNs treated with different concentration of glutamate and SMER28. Lysis buffer (LB) was used to evaluate the maximum LDH release (One-way ANOVA followed by Fisher's LSD test; ****p<0.0001). (D) The bar graph represents the quantification of the cell percentage with >3 GFP-LGALS3 puncta. M2-MNs were treated with LLoMe and/or SMER28; the fields were randomly selected and at least 25

cells for each sample were counted over 6 independent biological samples for each condition (n=6) ± SD (two-way ANOVA with Fisher's LSD test; *$p<0.05$; **$p<0.01$; ***$p<0.001$).

Supplementary Table 1 - List of antibodies used.

Antibody	Application	Dilution	Source
Mouse monoclonal anti-FLAG M2	WB<br>IF	1:1,000<br>1:500	Sigma-Aldrich, F1804
Mouse monoclonal anti-GAPDH	WB	1:3,000	Immunological Sciences, MAB-10578
rabbit polyclonal anti-Histone 3 (H3)	WB	1:30,000	Abcam, ab1791
rabbit polyclonal TFEB	WB<br>IF	1:4,000<br>1:500	Bethyl Laboratories, A303-673A
rabbit polyclonal anti-TFE3	WB<br>IF	1:1,000<br>1:500	Sigma-Aldrich, HPA023881
Mouse monoclonal anti-6xHIS	WB	1:1,000	Thermo Fisher, MA1-21315
Rabbit polyclonal anti-p62/SQSTM1	WB	1:2,000	Sigma-Aldrich, P0067
Rabbit polyclonal anti-LC3	WB	1:2,000	Sigma-Aldrich, L8918
Rabbit Monoclonal anti-VCP	WB<br>IF	1.1,000<br>1:500	Abcam, ab109240
Mouse monoclonal anti-polyGA	WB	1:1,000	Millipore; MABN889
Mouse Monoclonal anti-Nestin	IF	1:33	R&D MAB1259
Mouse Monoclonal anti-Islet	IF	1:50	Hybridoma, 40.2D6-s
Rabbit Monoclonal anti-B3Tubulin	IF	1:500	Cell signaling, 5568
Mouse monoclonal SMI32	IF	1.200	Calbiochem, NE1023
goat anti-mouse IgG-HRP	WB	1:5,000	Jackson ImmunoResearch, 115–035-003
goat anti-rabbit IgG-HRP	WB	1:5,000	Jackson ImmunoResearch, 111-035-003
Alexa Fluor® 594 goat anti-mouse IgG	IF	1:1,000	Thermo Fisher Scientific, A11020
Alexa Fluor® 594 goat anti-mouse IgG	IF	1:1,000	Thermo Fisher, A11020
Alexa Fluor® 594 goat anti-rabbit IgG	IF	1:1,000	Thermo Fisher Scientific, A11072

Supplementary Table 2 - List of primers used.

Target	Primer forward	Primer reverse
hp62/SQSTM1	5′ - CCA GAG AGT TCC AGC ACA GA - 3′	5′ -CCG ACT CCA TCT GTT CCT CA - 3′
hMAP1LC3B	5′ - CAG CAT CCA ACC CAA AAT CCC - 3′	5′ - GTT GAC ATG GTC AGG TAC AAG - 3′
hLAMP1	5’ – CGT GTC ACG AAG GCG TTT TCA G – 3’	5’ – CTG TTC TCG TCC AGC AGA CAC – 3’
hLAMP2A	5’ – GGC AAT GAT ACT TGT CTG CTG GC – 3’	5’ – GTA GAG CAG TGT GAG AAC GGG CA – 3’
hTFEB	5’ – CAA GGC CAA TGA CCT GGA C – 3’	5’ – AGC TCC CTG GAC TTT TGC AG – 3’
hHSPB8	5’ – AGA GGA GTT GAT GGT GAA GAC C – 3’	5’ – CTG CAG GAA GCT GGA TTT TC – 3’
hCTSB	5’ – GCT TCG ATG CAC GGG AAC ATT G – 3’	5’ – CAT TGG TGT GGA TGC AGA TCC G – 3’
hMNLCN1	5’ – CGG ACT GCT ATA CCT TCA GCG T – 3’	5’ – GGT GCT TAC ACT CCT GGA TGT G – 3’
hMNX1	5’ – GCC TAA GAT GCC CGA CTT CAA C – 3’	5’ – CGC GAC AGG TAC TTG TTG AGC T – 3’
hISL1	5’ – GCA GAG TGA CAT AGA TCA GCC TG – 3’	5’ – GCC TCA ATA GGA CTG GCT ACC A – 3’
hLHX4	5’ – TGG CGG ACA GGT GCT TCT CCA – 3’	5’ – AGG TGG TAG ACA AAG TCC TGG G – 3’
hRPLP0	5′ - GTG GGA GCA GAC AAT GTG GG - 3′	5′ - TGC GCA TCA TGG TGT TCT TG - 3′
mVcp	5’ – TGC CAT CCT AAA AGC CAA TC – 3’	5’ – TCA GCT CCA GAA AAG CCA TT – 3’
mRplp0	5′ – GGT GCC ACA CTC CAT CAT CA- 3′	5′ - AGG CCT TGA CCT TTT CAG TAA GT - 3′
