# Supplementary material for: VCP modulation ameliorates pathological features in C9orf72 models
Source: Cell Death Dis. 2026 May 17;17(1):629. doi: 10.1038/s41419-026-08856-1 (PMC13346533; doi:10.1038/s41419-026-08856-1)
Supplement: Supplementary file 2 — UNCROPPED_REVISED WB and FRA [file 41419_2026_8856_MOESM2_ESM.pptx]

## Slide 1
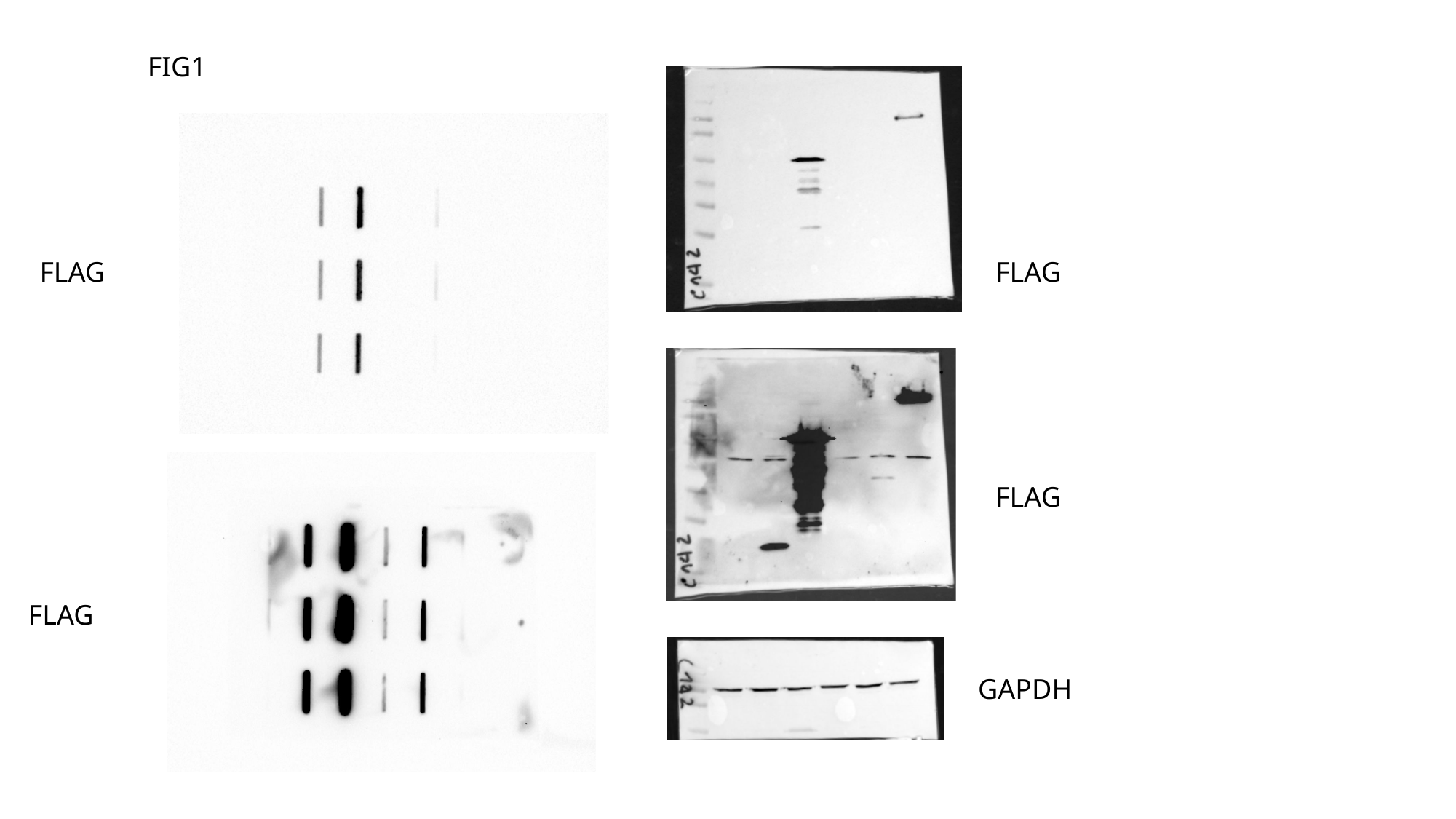

FIG1
FLAG
FLAG
FLAG
FLAG
GAPDH

## Slide 2
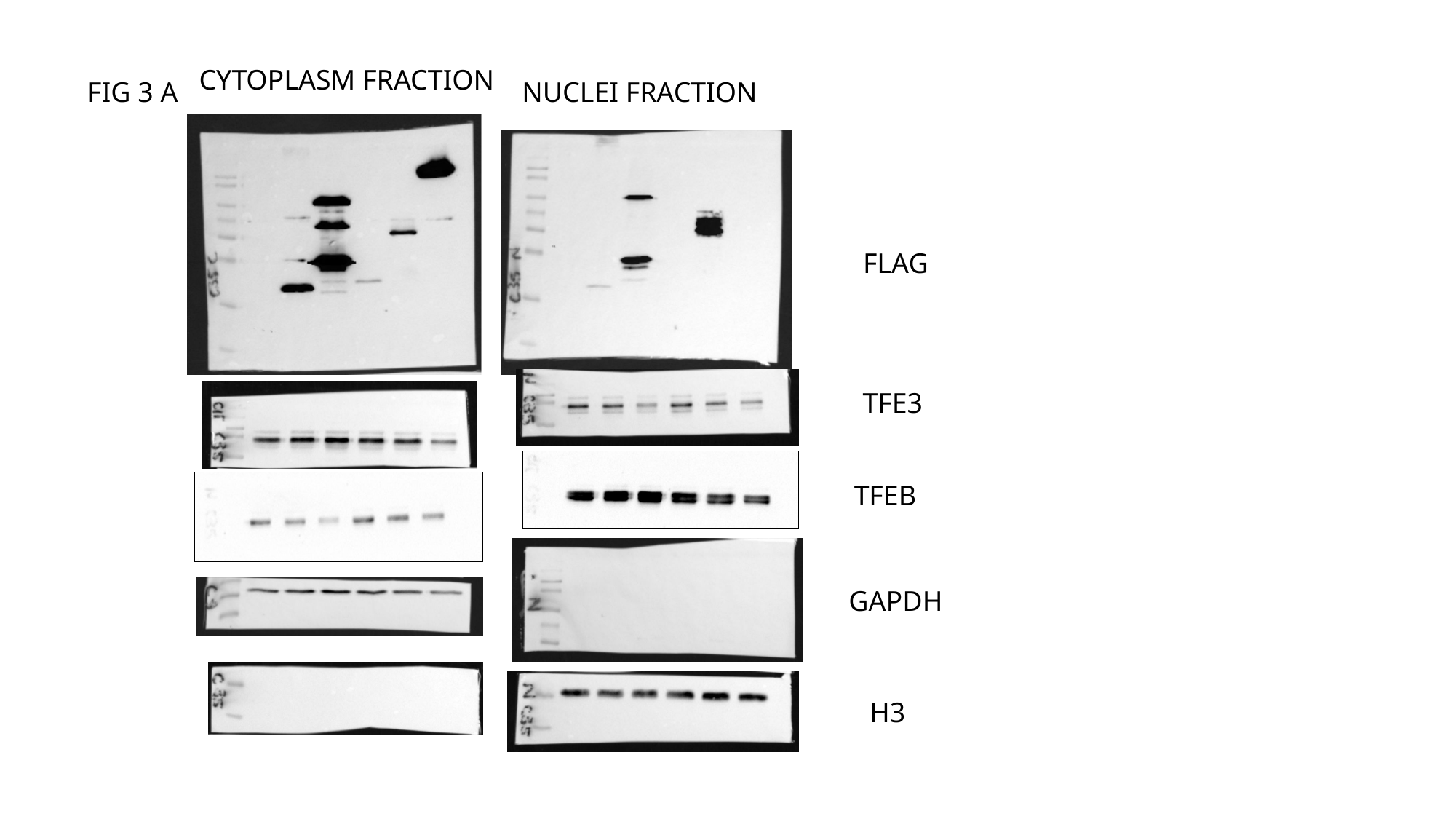

CYTOPLASM FRACTION
FIG 3 A
NUCLEI FRACTION
FLAG
TFE3
TFEB
GAPDH
H3

## Slide 3
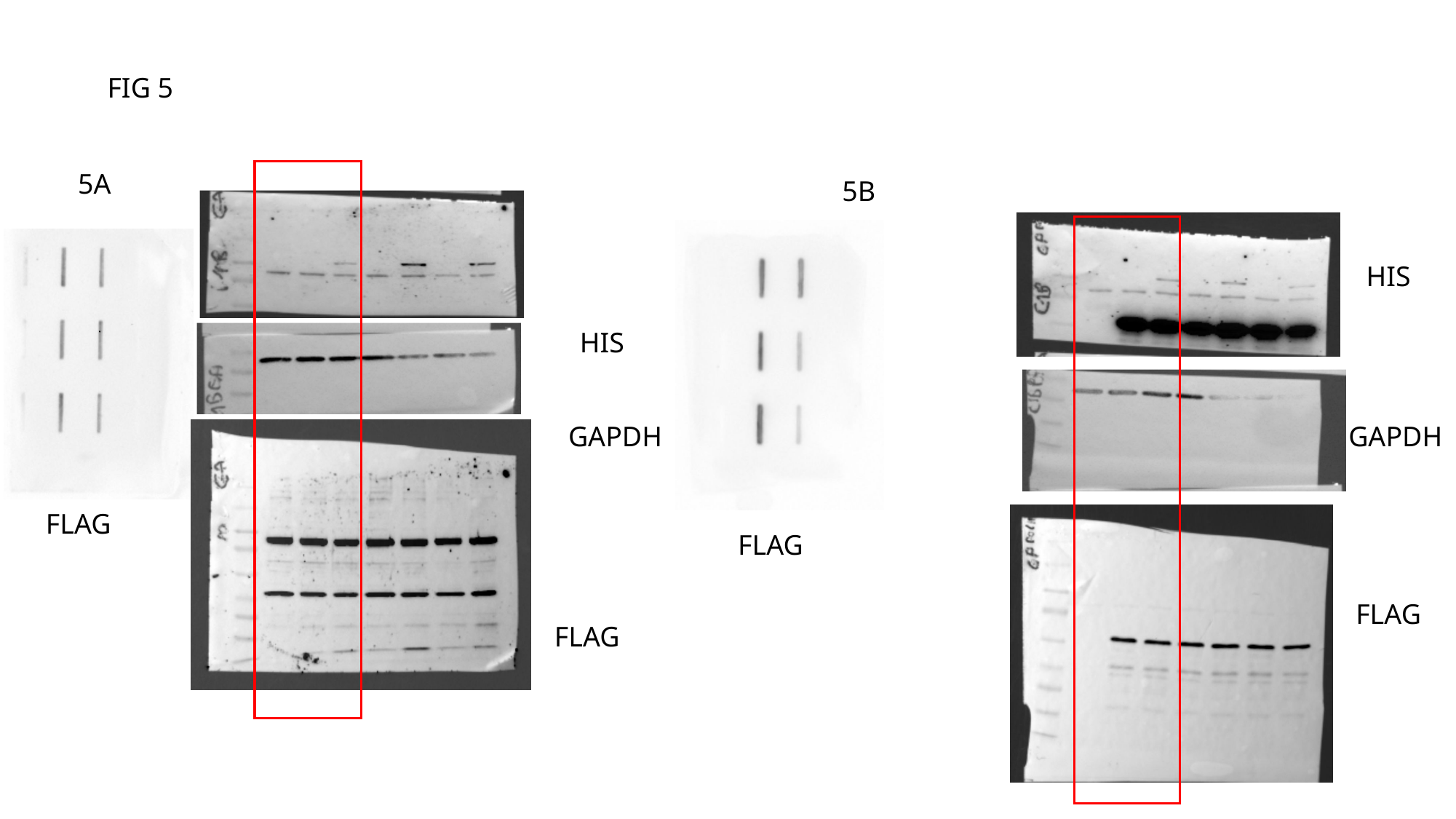

FIG 5
5A
5B
HIS
HIS
GAPDH
GAPDH
FLAG
FLAG
FLAG
FLAG

## Slide 4
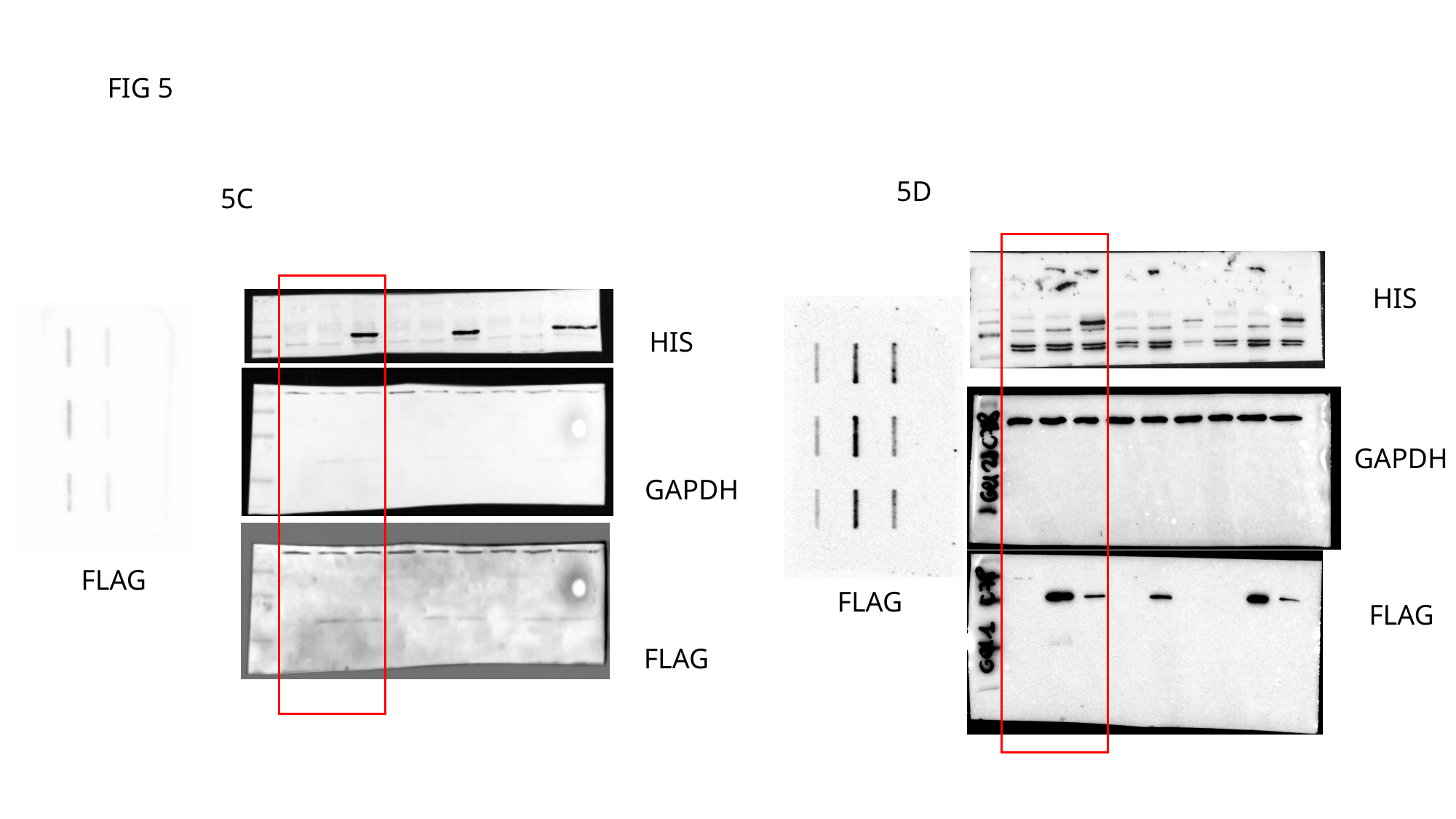

FIG 5
5D
5C
HIS
HIS
GAPDH
GAPDH
FLAG
FLAG
FLAG
FLAG

## Slide 5
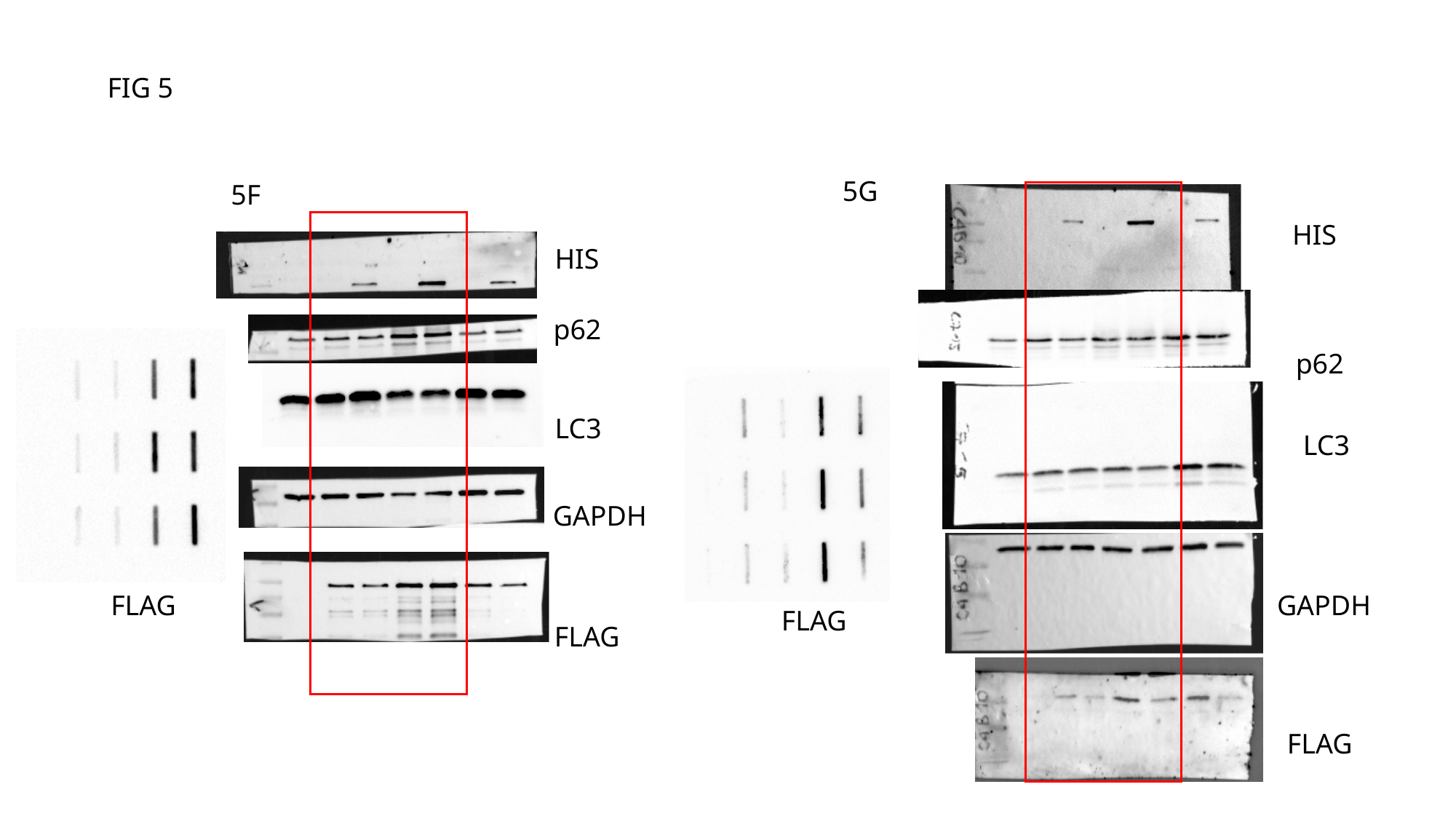

FIG 5
5G
5F
HIS
HIS
p62
p62
LC3
LC3
GAPDH
FLAG
GAPDH
FLAG
FLAG
FLAG

## Slide 6
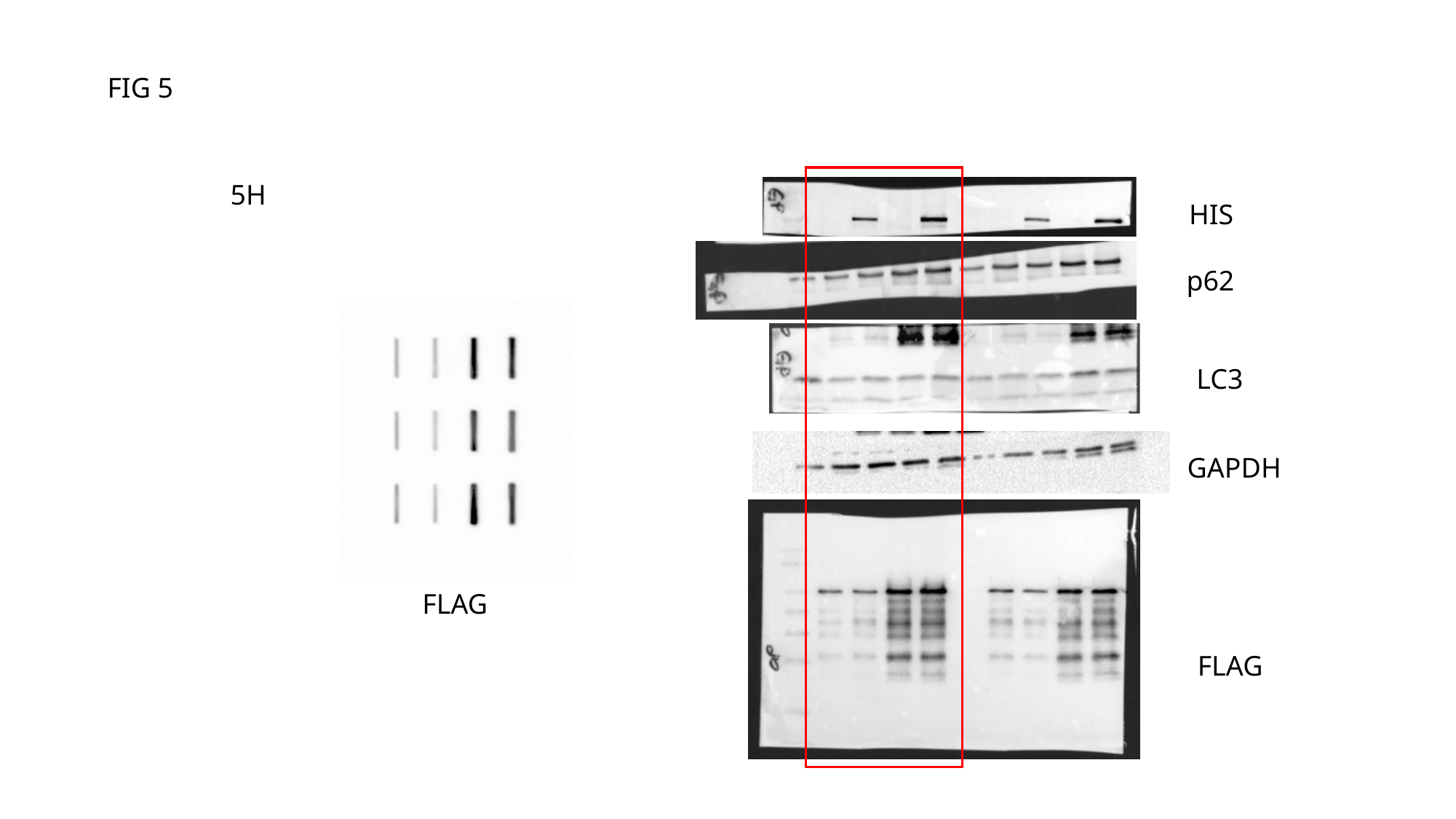

FIG 5
5H
HIS
p62
LC3
GAPDH
FLAG
FLAG

## Slide 7
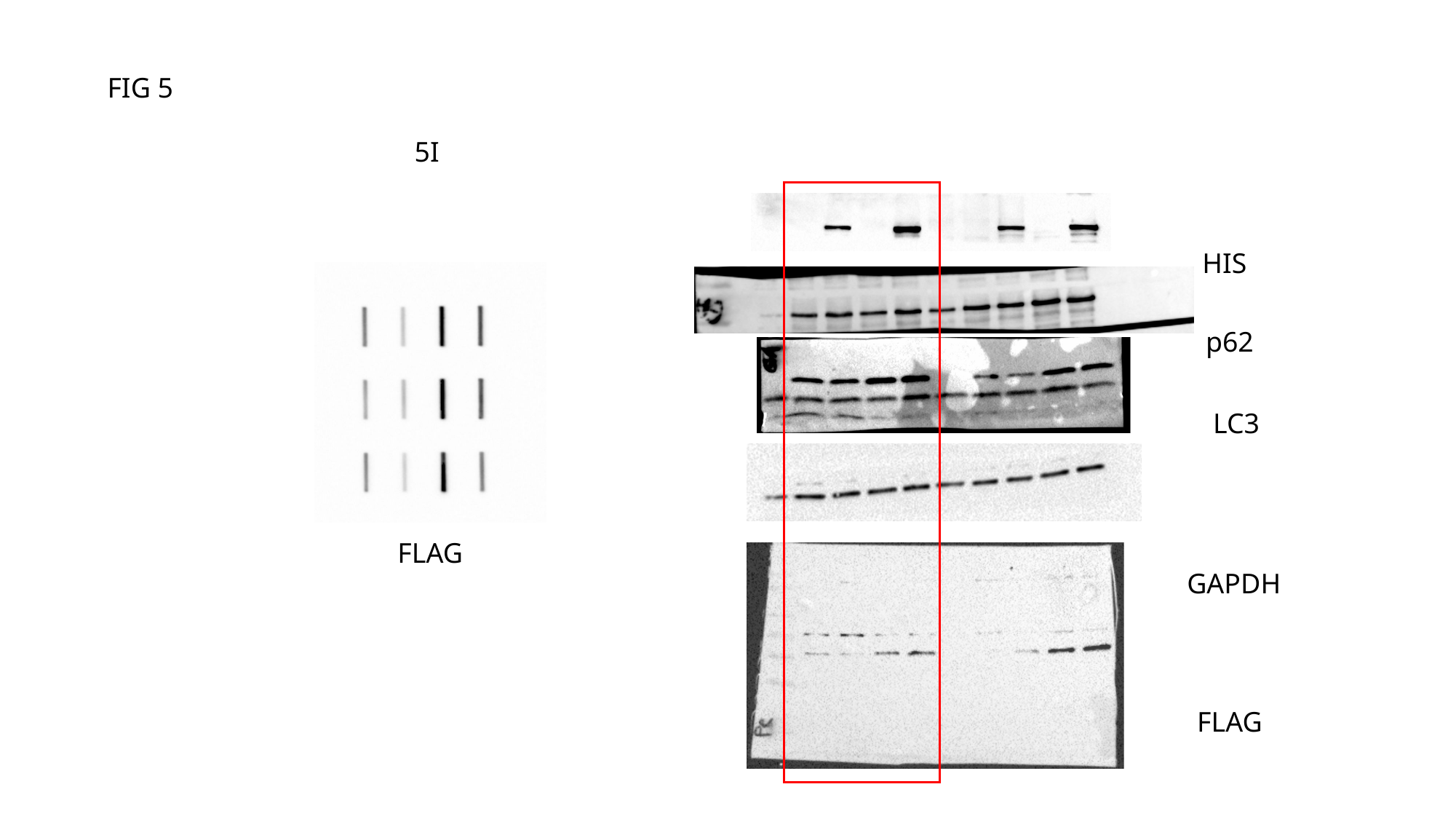

FIG 5
5I
HIS
p62
LC3
FLAG
GAPDH
FLAG

## Slide 8
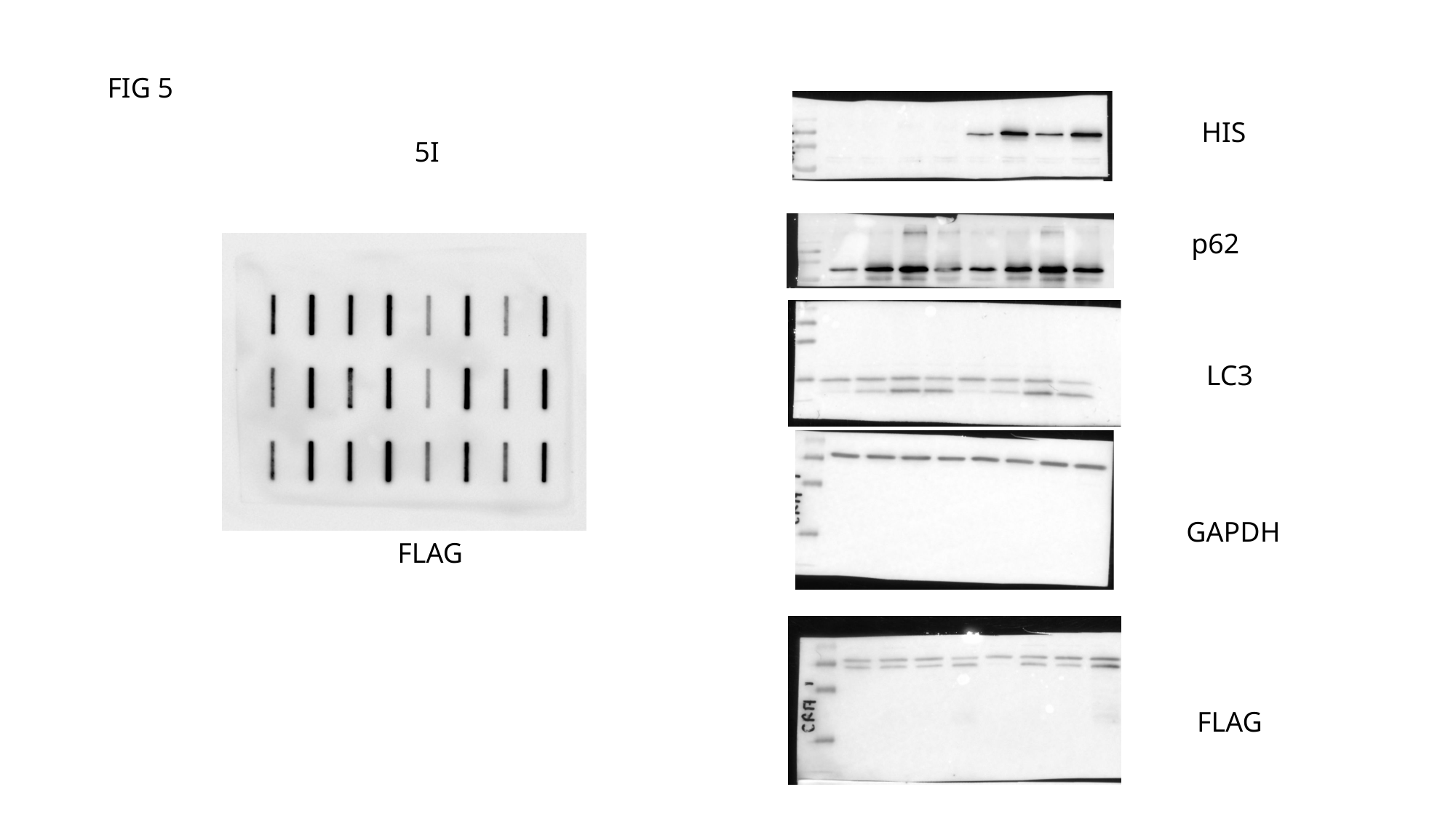

FIG 5
HIS
5I
p62
LC3
GAPDH
FLAG
FLAG

## Slide 9
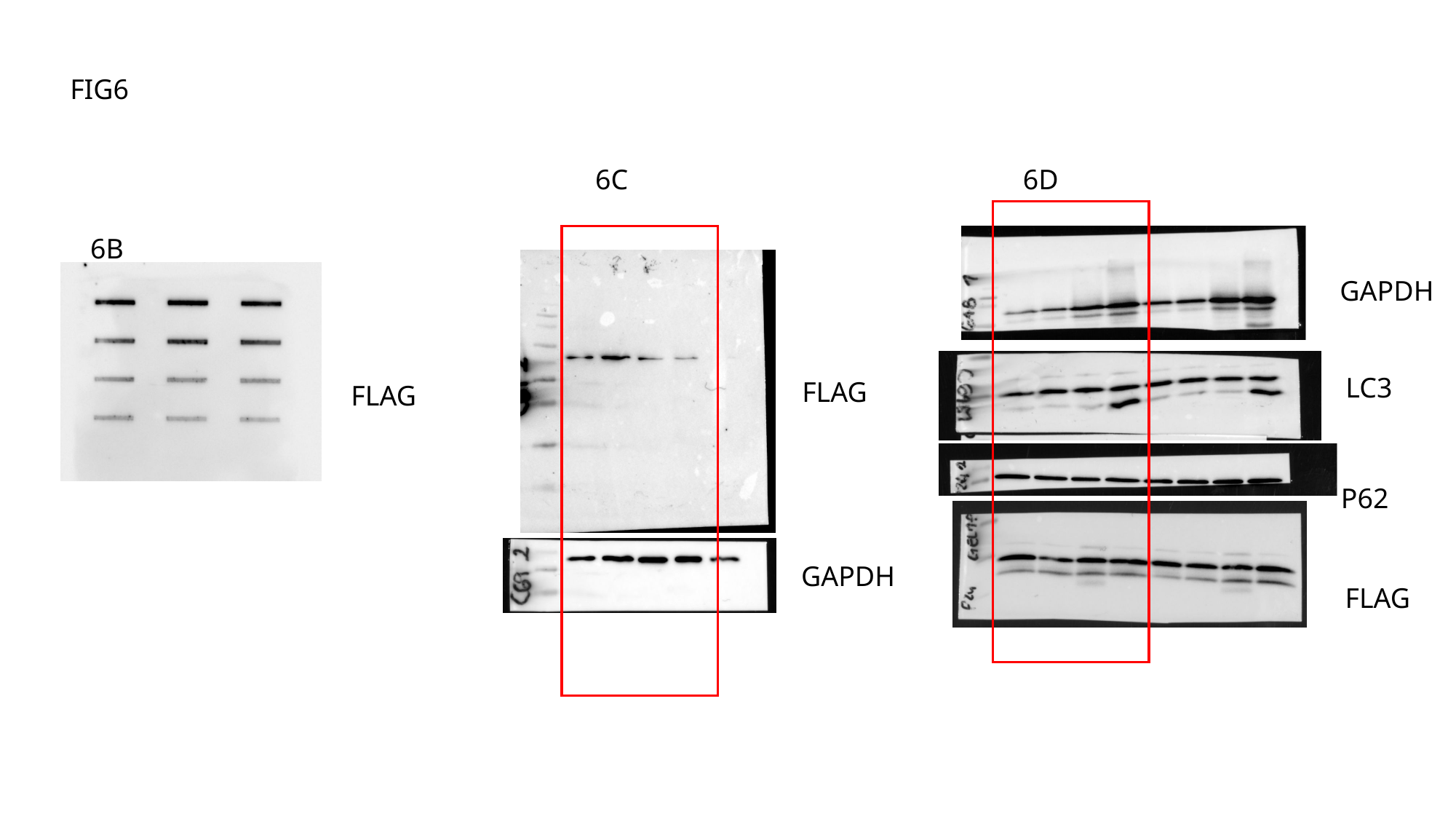

FIG6
6C
6D
6B
GAPDH
LC3
FLAG
FLAG
P62
GAPDH
FLAG

## Slide 10
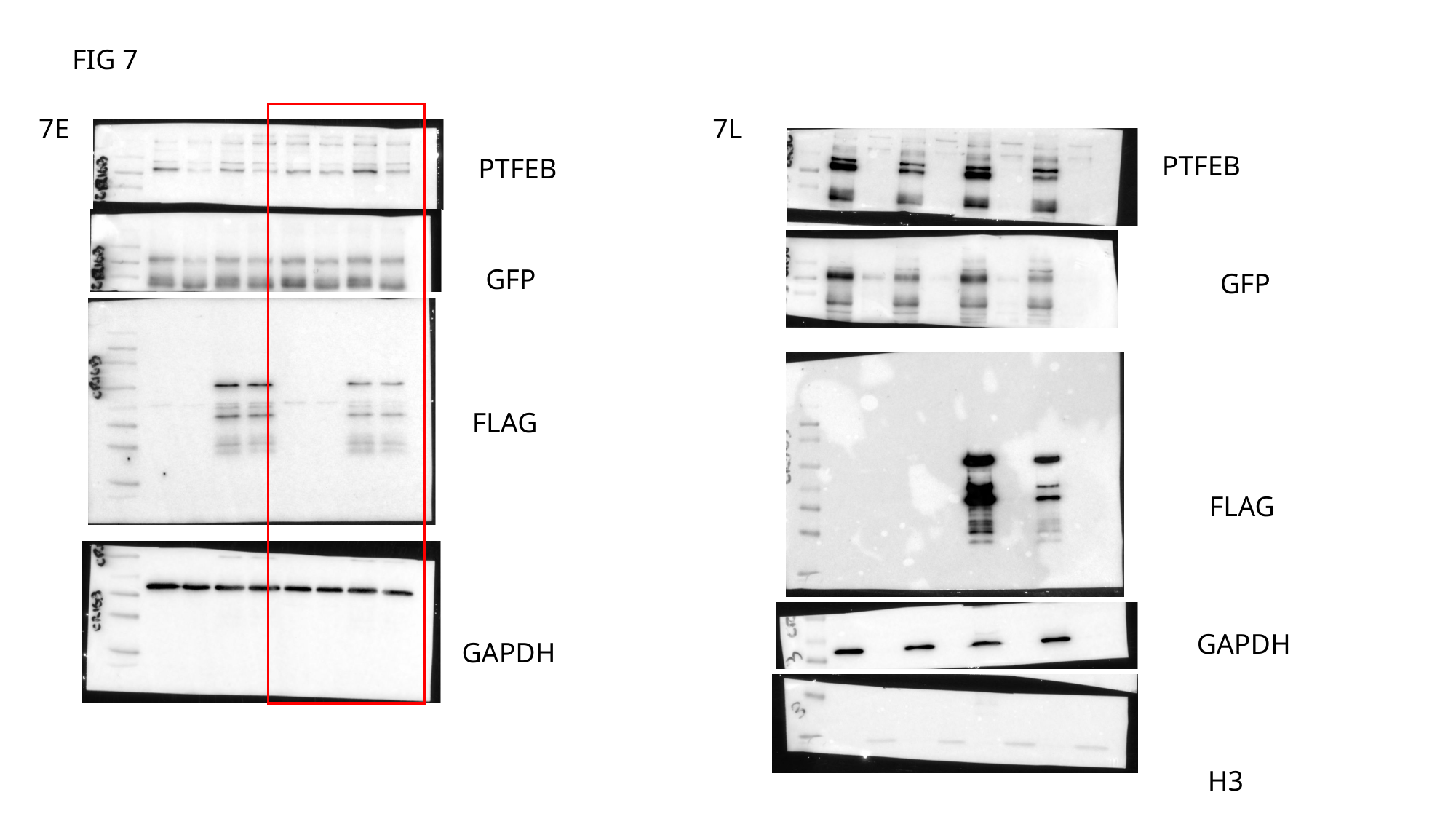

FIG 7
7E
7L
PTFEB
PTFEB
GFP
GFP
FLAG
FLAG
GAPDH
GAPDH
H3

## Slide 11
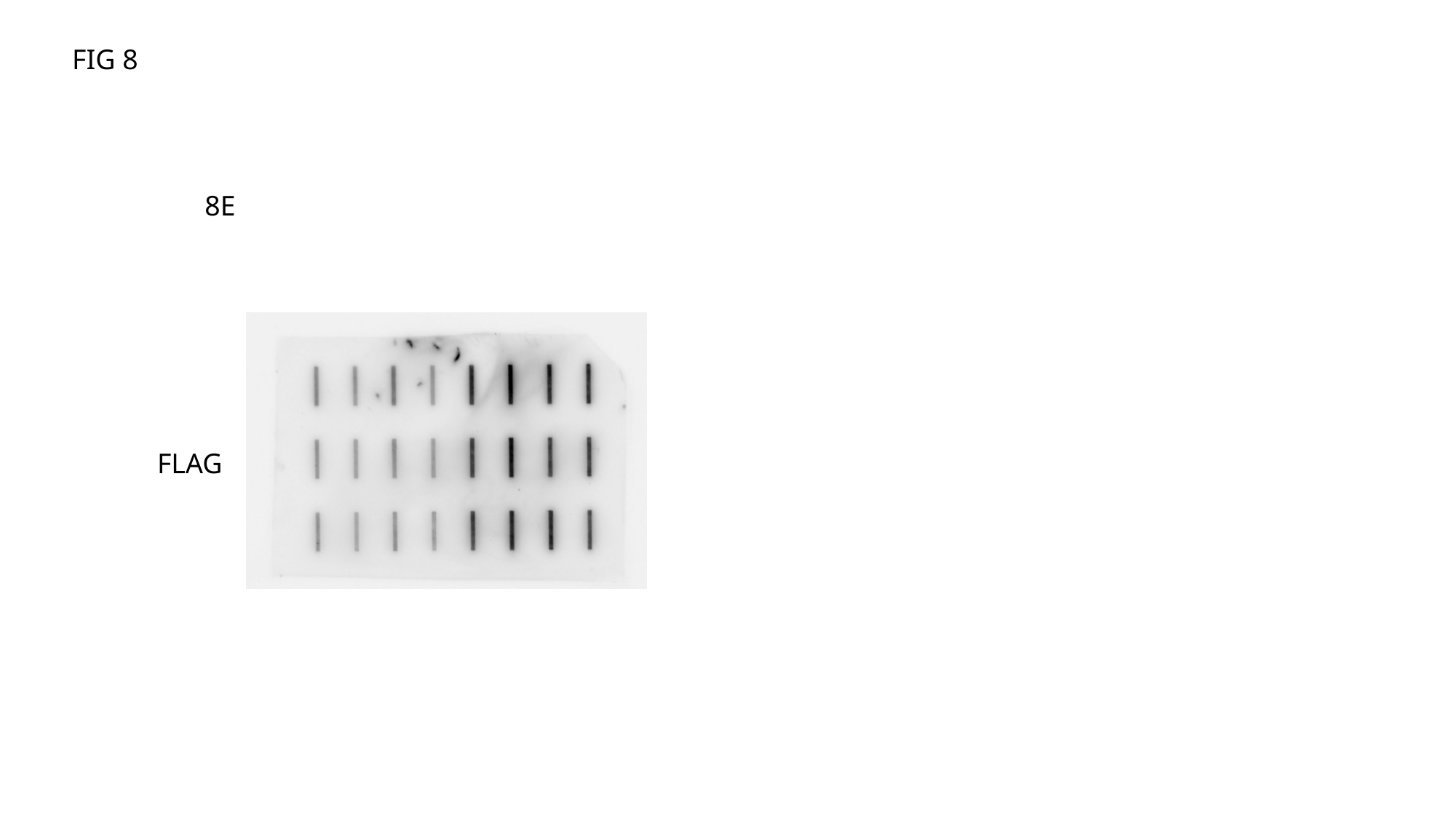

FIG 8
8E
FLAG
